# Systemic Oxidative Stress and Oxidized Albumin Mediate the Pathogenic Kidney-to-Gut Crosstalk by Disrupting Intestinal Barrier Integrity

**DOI:** 10.3390/biom16030462

**Published:** 2026-03-18

**Authors:** Jie Cheng, Yang Sui, Xin Wang, Yijun Xu, Rui Jiang, Yingyu Zhang, Zhuheng Shi, Mika Suda, Jianglin Fan, Jian Yao

**Affiliations:** Division of Molecular Signaling, Department of the Advanced Biomedical Research, Interdisciplinary Graduate School of Medicine, University of Yamanashi, Chuo City 409-3898, Japanjianglin@yamanashi.ac.jp (J.F.)

**Keywords:** oxidized albumin, kidney–gut axis, barrier dysfunction, microbiota, antioxidant therapy

## Abstract

Deleterious crosstalk between the gut and distant organs is a key factor behind disease progression. Currently, the molecular signals mediating this communication remain elusive. We hypothesized that systemic oxidative stress and oxidatively modified serum proteins transmit injury signals from extraintestinal sites to the gut. In various murine models of organ injury, primary damage was consistently associated with systemic oxidative stress and intestinal damage. Specifically, ischemia/reperfusion (I/R)-induced acute kidney injury caused profound colonic barrier defects. Depleting the microbiota with antibiotics markedly improved survival and attenuated both renal and colonic injury, implicating translocated microbes in exacerbating pathology. Mechanistically, these changes were linked to systemic oxidative stress and were largely prevented by the antioxidant N-acetylcysteine. Furthermore, serum from I/R mice disrupted epithelial barrier integrity and induced cell death in vitro, effects that were recapitulated by exposure to oxidized serum proteins. Characterization of serum components identified albumin as the predominantly oxidized protein, which displayed potent cytotoxicity toward cultured intestinal epithelial cells. Our findings establish oxidative stress and oxidized serum albumin as key pathogenic factors mediating the detrimental interaction between remote organs and the gut. These data suggest that targeting oxidative modifications offers a promising therapeutic strategy to disrupt this pathological loop in critical illness.

## 1. Introduction

Inter-organ communication is a fundamental biological process essential for maintaining systemic homeostasis. Among various interactive networks, the gut–organ axis is particularly important. Under pathological conditions, the dysfunction of the crosstalk has been recognized as a critical factor contributing to disease progression. In chronic kidney disease (CKD), for example, gut dysbiosis promotes the accumulation of uremic toxins, such as indoxyl sulfate, which enter the circulation and accelerate renal fibrosis and cardiovascular pathology [1,2,3]. Similarly, in sepsis, compromised intestinal barrier integrity permits the translocation of endotoxins, causing systemic inflammatory response syndrome (SIRS) and multi-organ failure [4]. In these scenarios, the gut acts as a potent amplifier of systemic inflammation and injury.

Conversely, primary injury in extraintestinal organs can reciprocally induce gut dysfunction. Acute heart failure, for instance, triggers splanchnic hypoperfusion and mucosal ischemia [5], while severe pancreatitis releases inflammatory cytokines and digestive enzymes that directly compromise the intestinal epithelium [6]. Emerging evidence suggests that this bidirectional communication creates a vicious loop in which the initial organ injury compromises the gut, and the failing gut, in turn, exacerbates the primary injury [7,8,9]. This cycle is a hallmark of multiple organ dysfunction syndrome (MODS), a condition associated with high mortality [10]. While the “gut-to-organ” pathway has been extensively investigated and documented [11,12,13], the mechanisms by which injury signals are transmitted from distant organs to the gut remain poorly understood. Identifying these initiating mediators is critical for disrupting the progression of critical illness.

Systemic oxidative stress is a common pathological feature of both acute and chronic organ injury. It is caused by an imbalance between reactive oxygen species (ROS) production and endogenous antioxidant defenses [14,15]. Generated by mitochondrial respiration or NADPH oxidases (NOX), excessive ROS damages macromolecules, activates redox-sensitive cell death pathways, and triggers inflammatory responses. Consistent with this, we have reported that organ injuries induced by various insults are associated with systemic oxidative stress. Antioxidant therapies are effective in preventing disease progression [16,17,18,19,20]. Furthermore, we have demonstrated that supplementing mice with vitamin C and copper—which generates massive ROS via the Fenton reaction—is sufficient to trigger systemic oxidative stress and multi-organ injury [16]. These findings implicate oxidative stress as a common mechanism in various pathologies and suggest it may play a central role in pathological inter-organ communication.

However, the mechanism by which localized oxidative stress propagates to remote organs remains an open question. Due to their high reactivity and short half-lives, ROS are unlikely to function as long-range endocrine signals [21]. Instead, the oxidatively modified serum proteins, which have a long half-life, could act as stable mediators of oxidative damage, transmitting a damaging signal. Among various serum proteins, albumin could be the leading candidate. As the most abundant plasma protein, albumin’s cysteine-34 (Cys34) residue constitutes approximately 80% of the circulating free thiol pool, serving as the major extracellular antioxidant trap. Under severe oxidative stress, this residue is highly susceptible to oxidation, leading to oxidized albumin (ox-Alb). Elevated levels of ox-Alb correlate with disease severity and mortality in sepsis, kidney failure, and heart disease [22,23]. Recent evidence suggests that ox-Alb is not merely a passive biomarker but an active pathogenic effector capable of inducing cellular senescence, inflammation, and direct cytotoxicity [24,25,26]. Our group has previously shown that ox-Alb exacerbates kidney disease by inducing ferroptosis in tubular cells [27], and others have noted its potential to damage intestinal epithelium [26]. Based on this, we hypothesized that ox-Alb functions as a signal carrier, transmitting oxidative injury from extraintestinal sites to the gut, thereby disrupting barrier integrity and aggravating systemic pathology. The purpose of this study was to address this hypothesis. Using a renal ischemia/reperfusion (I/R) model of acute kidney injury (AKI), we demonstrate that systemic oxidative stress and ox-Alb contribute to intestinal barrier dysfunction, promoting microbial translocation and worsening systemic conditions. Our study thus characterizes ox-Alb as a key pathogenic factor in organ–gut crosstalk. It suggests that targeting oxidized plasma proteins could be developed as a promising therapeutic strategy to halt the progression of multi-organ dysfunction.

## 2. Materials and Methods

### 2.1. Materials

Sodium dextran sulfate 5000 (DSS, cat no. 199-08361) was purchased from Wako Pure Chemical Corporation (Tokyo, Japan). Doxorubicin (DOX, cat no. D4193) and 2,4,6-trinitrobenzenesulfonic acid (TNBS, cat no. T1340) were obtained from Tokyo Chemical Industry (Tokyo, Japan), and pentobarbital sodium (cat. no. 0514101) was obtained from Kyoritsu Seiyaku Corporation (Tokyo, Japan). A blood urea nitrogen (BUN, cat no. ELABUN) kit was purchased from Thermo Fisher Scientific (Frederick, MD, USA). Creatinine (cat no. 700460) was from Cayman Chemical Company (Ann Arbor, MI, USA). Anti-podocin (cat no. sc-21009) and Anti-E-Cadherin (cat no. sc-7870) were bought from Santa Cruz Biotechnology (Santa Cruz, CA, USA). Anti-lipocalin-2 antibody (cat no. AG-25A-0088) was acquired from Adipogen (San Diego, CA, USA). Anti-ACSL4 (cat no. 2401-1-AP) and anti-albumin (cat no. 164751-AP) antibodies were obtained from Proteintech Genomics (San Diego, CA, USA), and Anti-β-actin (cat no. A5316) was supplied by Sigma-Aldrich (St. Louis, MO, USA). Anti-xCT (cat no. NB300-317) antibodies were purchased from bio-techne (Minneapolis, MN, USA). Alexa 680 Fluor C2 maleimide (cat no. A20344) was from Thermo Scientific (Rockford, IL, USA). Bovine serum albumin (BSA, Fraction V, cat no. A001) was purchased from Iwai Chemical Company (Tokyo, Japan). Calcein-AM and Propidium Iodide (PI) Staining Kit (cat no. A20344) was sourced from Dojindo Molecular Technologies (Rockville, MD, USA). Minute^TM^ albumin depletion reagent (cat no. WA-013) was sourced from Invent Biotechnologies, Inc. (Plymouth, MN, USA). Hydrogen Peroxide (H_2_O_2,_ cat no. 084-07441) was sourced from Fujifilm (Tokyo, Japan). FITC-dextran (cat no. NB300-317), glutathione (GSH, cat no. G257), OxyBlot^TM^ Protein Oxidation Detection Kit (cat no. S7150), acetaminophen (APAP, cat no. 103-90-2), L-Ascorbic acid (AA, cat no. A5960), N-Acetyl-L-cysteine (NAC, cat no. A9165), Sodium hydrosulfide hydrate (NaHS, cat no.161527) and all other chemicals were acquired from Sigma (St. Louis, MO, USA).

### 2.2. Experimental Animals and Animal Procedures

Adult male C57BL/6 mice (25–35 g, 10–13 weeks old) were housed under standard conditions with controlled room temperature, a 12-h light/dark cycle, and free access to standard rodent chow and water. All animal experiments were approved by the Institutional Animal Care and Use Committee of Yamanashi University (Approval No. A3-583) and conducted in strict accordance with the institutional guidelines for the care and use of laboratory animals.

The DOX-induced cardiotoxicity model was established as previously reported [20]. Briefly, mice were intraperitoneally injected with 25 mg/kg DOX or an equivalent volume of normal saline as a control. Blood and tissues were collected 3 days post-injection.

The APAP-induced acute liver injury model was also induced, as we have reported [19]. Mice were injected intraperitoneally with 500 mg/kg APAP or an equivalent volume of saline as a control. Blood and tissues were collected 24 h post-injection. Therapeutic intervention with sulfhydrylated albumin (Alb-SSH) was performed as we have previously reported. Briefly, Alb-SSH (1 g/kg) was administered intraperitoneally at an interval of 12 h for 4 times before DOX or APAP injection.

The I/R-induced AKI model was established as previously reported, with minor modifications [17]. Mice were randomly assigned to sham, renal I/R, antibiotic intervention, or N-acetylcysteine (NAC) intervention groups, normally each with 3~4 mice per group. For mice mortality with or without antibiotic intervention, the mice number was 6. Mice were anesthetized via intraperitoneal injection (IP) of sodium pentobarbital and placed on a heating pad to maintain body temperature at 37 °C. Following bilateral flank incisions, the renal pedicles were isolated and occluded with non-traumatic microvascular clips for 45 min. Reperfusion was initiated by removing the clips. Sham-operated mice underwent the same surgical procedure without pedicle clamping. Twenty-four hours after reperfusion, mice were euthanized for sample collection.

Gut microbiota depletion was achieved using the protocol described by Kelly et al. [28], as detailed in our previous report [18]. Briefly, mice were administered a broad-spectrum antibiotic cocktail containing ampicillin (1 mg/mL), neomycin (1 mg/mL), metronidazole (1 mg/mL), vancomycin (0.5 mg/mL), and gentamicin (1 mg/mL) via oral gavage (10 μL/g body weight) every 12 h for four consecutive days before I/R surgery. Antioxidant therapy was performed as previously described [16]. Briefly, NAC (200 mg/kg) was administered intraperitoneally every 12 h for 3 days before I/R surgery.

The animal experiments presented in this study were conducted as multiple independent cohorts at different time points. To ensure the internal validity of our findings, all experimental and control groups within a single cohort were age- and weight-matched and processed concurrently. All statistical analyses were performed exclusively between groups within the same cohort; no inter-cohort comparisons were made.

### 2.3. Cell Culture

The human colon adenocarcinoma cell line Caco-2 was cultured in DMEM/F12 medium supplemented with 5% fetal bovine serum and 1% antibiotics and maintained in a humidified incubator at 37 °C with 5% CO_2_. For the experiments, cells were seeded in medium containing 0.5% fetal bovine serum. Upon reaching confluence, cells were subjected to the indicated experimental treatments.

### 2.4. Western Blot Analysis

Western blotting was performed as previously described [18,20]. Total protein was extracted from cells and tissues using RIPA lysis buffer containing protease inhibitors. Protein concentration was determined using the BCA method. Equal amounts of protein were separated by 10–12% SDS-PAGE and transferred onto PVDF membranes. Membranes were blocked with 5% skim milk or 1% BSA for 1 h at room temperature, followed by incubation with primary antibodies at 4 °C overnight. Primary antibodies used included: β-actin (1:2000), lipocalin-2 (1:3000), E-cadherin (1:3000), albumin (1:1000), ACSL4 (1:6000), xCT (1:3000), podocin (1:1000), and protein carbonylation antibody (1:150). After washing with PBST, the blots were incubated with a peroxidase-conjugated secondary antibody (1:3000), or protein carbonylation secondary antibody (1:300) for 1 h, and the bands of the target proteins were detected using an enhanced chemiluminescence system (Nacalai Tesque, Kyoto, Japan). The chemiluminescent signal was captured with a Fujifilm Image LAS-4000 analyzer (Fujifilm, Tokyo, Japan), and band intensities were quantified with NIH ImageJ, version 1.51k (https://imagej.net/ij/, accessed on 23 February 2017).). β-actin or EZ-Blue staining was used as an internal control to ensure equal loading of the sample protein.

### 2.5. Detection of Free Sulfhydryl (-SH) Groups Using Maleimide Assay

The levels of free sulfhydryl (-SH) groups in serum, intestinal, and kidney tissues were determined using the maleimide labeling assay as previously reported [19,20]. Briefly, proteins from serum, intestinal, and kidney tissues were incubated with 5 μM Alexa Fluor 680 C2 Maleimide (Thermo Scientific, Rockford, IL, USA) for 2 h at 4 °C. The labeled proteins were then separated by SDS-PAGE, and the fluorescence signals in the bands were captured using a Fujifilm Las-4000 imaging system (Fujifilm, Tokyo, Japan).

### 2.6. Assessment of Protein Carbonylation

Protein carbonylation was analyzed using the OxyBlot Protein Oxidation Detection Kit (EMD Millipore, Billerica, MA, USA) according to the manufacturer’s instructions, as previously reported [27]. Briefly, mouse serum diluted at 1:20 or protein lysates from cells/tissues were extracted using SDS lysis buffer (62.5 mM Tris-HCl, 2% SDS, 10% glycerol) containing 1% proteinase inhibitor cocktail (Nacalai Tesque, Kyoto, Japan) and 50 mM DTT. Five microliters of protein sample (5–10 μg) were mixed with 5 μL of 12% SDS and 10 μL of 2,4-dinitrophenylhydrazine solution and incubated for 15 min at room temperature for denaturation and derivatization. After the reaction, samples were neutralized with 7.5 μL of neutralization solution and subjected to Western blot analysis.

### 2.7. Serum Albumin Depletion

Serum albumin was depleted using a commercially available albumin depletion reagent according to the manufacturer’s instructions (MinuteTM albumin depletion reagent; cat no.WA-013; Invent Biotechnologies, Inc.; Plymouth, MN, USA). Briefly, 50 μL of mouse serum was thoroughly mixed with an equal volume of the albumin depletion reagent. The mixture was centrifuged at 13,200 rpm for 5 min, and the supernatant containing depleted albumin was discarded. The remaining pellet was collected, resuspended, and used for subsequent experiments.

### 2.8. Preparation of Sulfhydrylated Albumin

Alb-SSH was prepared as we previously reported [19]. The process involved exposure of -SH groups in albumin with dithiothreitol (DTT) treatment, followed by formation of -SOH group with H_2_O_2_ treatment and subsequent replacement of -SOH with H_2_S donor NaHS to form -SSH-Alb [29]. Briefly, bovine albumin at 250 mg/mL in distilled water was exposed to 100 mM DTT at 4 °C overnight, which was followed by exhaustive dialysis against distilled water or 0.9% sodium chloride using 7000 MWCO dialysis tubing (Thermo Scientific). A portion of the DTT-treated protein was further modified by reacting with 1 mM H_2_O_2_ at room temperature for 1 h to form -SOH. The -SOH groups were then allowed to incubate with NaHS at 4 °C overnight to form -SSH groups. The unreacted chemicals were removed through exhaustive dialysis. After the determination of protein concentration and confirmation of -SSH group formation, as well as its antioxidative activities, as described in our previous report [19] the sulfhydrylated albumin was aliquoted and stored at -80 °C until use.

### 2.9. Preparation of Oxidized Serum and Albumin

Oxidized proteins were generated via a copper-catalyzed Fenton reaction, as previously described [16,27]. For ox-Serum, mouse serum was diluted 1:1 with PBS and incubated with 0.4 M Ascorbic Acid (AA) and 2 mM CuCl_2_ at 37 °C for 2 h. For ox-Alb, BSA (40 mg/mL in PBS) was incubated with 0.4 M AA and 2 mM CuCl_2_ at 4 °C for 48 h. Following incubation, low-molecular-weight reactants were removed. Ox-serum was purified using Amicon Ultra centrifugal filters (Millipore), and ox-Alb was extensively dialyzed against PBS using a dialysis cassette (MWCO 7 kDa; Thermo Fisher Scientific). The oxidative status of the resulting proteins was confirmed by assessing free -SH levels and protein carbonylation.

### 2.10. WST Assay

WST assay was performed as we have previously reported [19]. Briefly, cells in 96-well plates were treated as indicated. WST reagent was added to each well, and the mixture was incubated for 30 min. Absorbance was measured at 450 nm using a spectrometer.

### 2.11. Calcein-AM and Propidium Iodide (PI) Staining

Live and dead cells were visualized using a Calcein-AM/PI staining kit according to the manufacturer’s instructions (Dojindo). Cells were incubated with a mixture of Calcein AM and PI at 37 °C for 10–20 min. The images of Calcein-AM-positive green live cells and PI-positive red dead cells were visualized and captured using a fluorescence microscope.

### 2.12. Measurement of Blood Urea Nitrogen (BUN) and Creatinine

BUN and creatinine levels were measured using commercial kits following the manufacturers’ protocols (Thermo Fisher Scientific; Frederick, MD, USA). Briefly, sera with appropriate dilution were reacted with the assay buffer. Optical absorption was measured at 450 nm for BUN and 490 nm for creatinine using a spectrometer (SpectraMax 340, Sunnyvale, CA, USA).

### 2.13. GSH Assay

GSH level was measured using a commercial assay kit as described previously [19]. Samples prepared in 0.05% sulfosalicylic acid (SSA) were mixed with the reaction buffer at 37 °C for 1 h. Afterward, coenzyme and substrate solutions were added for an additional 30 min. Absorbance was measured at 405 nm using a spectrophotometer. Concentrations of GSH were calculated based on the standard curves generated from the GSH standard.

### 2.14. Permeability Assays

For in vivo gut permeability, renal I/R surgery was performed as described above and gut permeability was determined using FITC-dextran as a probe. At 20 h post-renal I/R surgery, the mice were orally gavaged with FITC-dextran (4 kDa, 40 mg/kg body weight in PBS). Four hours later (24 h post-surgery), blood was collected, and serum was isolated. The concentration of FITC-Dextran in the serum was determined via the intensity of fluorescent signal captured by a SpectraMax^®^ GEMINI EM (Sunnyvale, CA, USA) at an excitation wavelength (λ) of 485 nm and an emission wavelength (λ) of 535 nm.

For in vitro monolayer permeability, Caco-2 cells were seeded at a density of 1.5 × 10^5^ cells per well in Transwell inserts (Millipore) in medium containing 0.5% fetal bovine serum until a confluent monolayer formed. Cells were then treated as indicated and exposed to FITC-Dextran (1 mg/mL) at the apical chamber. After 24 h, samples were collected from the basolateral chamber, and fluorescence was measured as described above.

### 2.15. Statistical Analysis

Values are expressed as mean ± standard error (SE). Comparisons between two groups were performed using an unpaired, two-tailed Student’s t-test. Comparisons among three or more groups were performed using one-way or two-way analysis of variance (ANOVA), followed by Tukey’s or Bonferroni’s multiple comparisons test, respectively. All analyses were conducted using Microsoft Excel or GraphPad Prism version 8.0 (GraphPad Software, San Diego, CA, USA). A *p*-value of less than 0.05 was considered statistically significant.

## 3. Results

### 3.1. Systemic Oxidative Stress and Remote Gut Injury Are Common Features of Diverse Organ Pathologies

**Figure 1:**
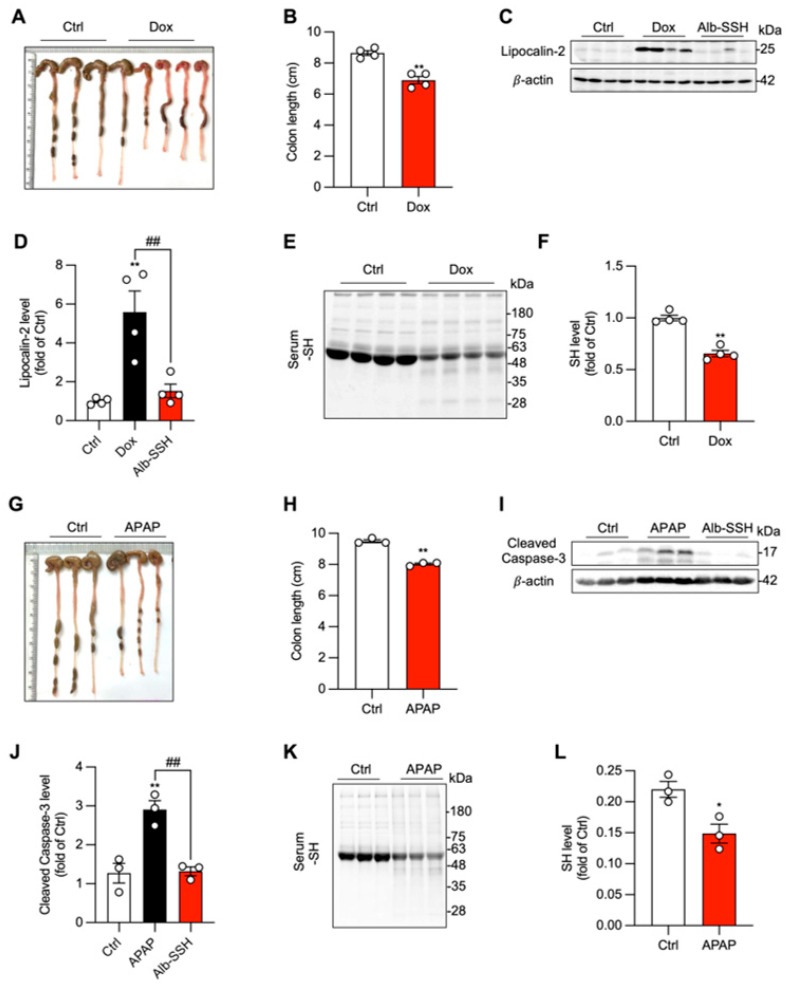
Systemic oxidative stress and remote gut injury are common features of diverse organ pathologies. (**A**,**B**) Mice were intraperitoneally injected with 25 mg/kg doxorubicin (DOX). After 24 h, colon length was photographed and measured. Quantitative results are presented in (**B**). (**C**,**D**) Protein level of the injury marker lipocalin2 in colon lysates was determined by Western blot. A representative blot is shown in (**C**), with quantitative data in (**D**). Note the complete prevention of the appearance of injury marker in mice treated with antioxidative sulfhydrylated albumin (Alb-SSH). (**E**,**F**) Serum protein sulfhydryl (-SH) groups were analyzed at 24 h after DOX administration. A representative blot is shown in (**E**). Quantitative analysis of the signal intensity at ~66 kDa (serum albumin) is presented in (**F**). (**G**,**H**) Colon length was photographed and measured at 24 h after acetaminophen (APAP, 500 mg/kg) administration. Quantitative results are presented in (**H**). (**I**,**J**) Protein level of the injury marker cleaved caspase-3 in colon lysates was determined by Western blot. A representative blot is shown in (**I**), with quantitative data in (**J**). (**K**,**L**) Serum protein -SH groups were analyzed at 24 h after APAP administration. A representative blot is shown in (**K**). Quantitative analysis of the signal intensity at ~66 kDa is presented in (**L**). Data shown are mean ± SE (*n* = 4 for (**B**,**D**,**F**) and *n* = 3 for (**H**,**J**,**L**); * *p* < 0.05, ** *p* < 0.01 vs. Ctrl; ^##^ *p* < 0.01). Original images of Western Blotting can be found in Supplementary Materials.

Pathological crosstalk between organs is a well-recognized phenomenon in critical illness. In our recent studies on DOX-induced cardiotoxicity and APAP-induced hepatotoxicity [19,20], we unexpectedly found that primary organ damage in the heart and liver was accompanied by remote gut injury, evidenced by colon shortening and the appearance of the injury marker lipocalin-2 or cleaved caspase-3 (Figure 1A–D, G–J). The phenomenon was associated with serum protein oxidation, as revealed by a significant reduction in serum protein -SH groups, especially at MW around 66 kDa (Figure 1E,F, K,L), the predicted molecular weight of serum albumin. Supplementation of mice with Alb-SSH (a modified form of serum albumin where hydrogen sulfide or sulfur groups are added to the free Cys34 residues of albumin), completely abolished the appearance of injury markers (Figure 1C,D,I,J). Of note, Alb-SSH has been characterized to have an elevated -SSH group, release H_2_S under the reductive conditions, and possess strong anti-oxidative activity in our previous reports [19,20]. These observations thus indicate that systemic oxidative stress might be a mechanistic link among pathological organ crosstalk.

### 3.2. Renal Ischemia/Reperfusion Induces Acute Kidney Injury and Remote Colonic Barrier Dysfunction

**Figure 2:**
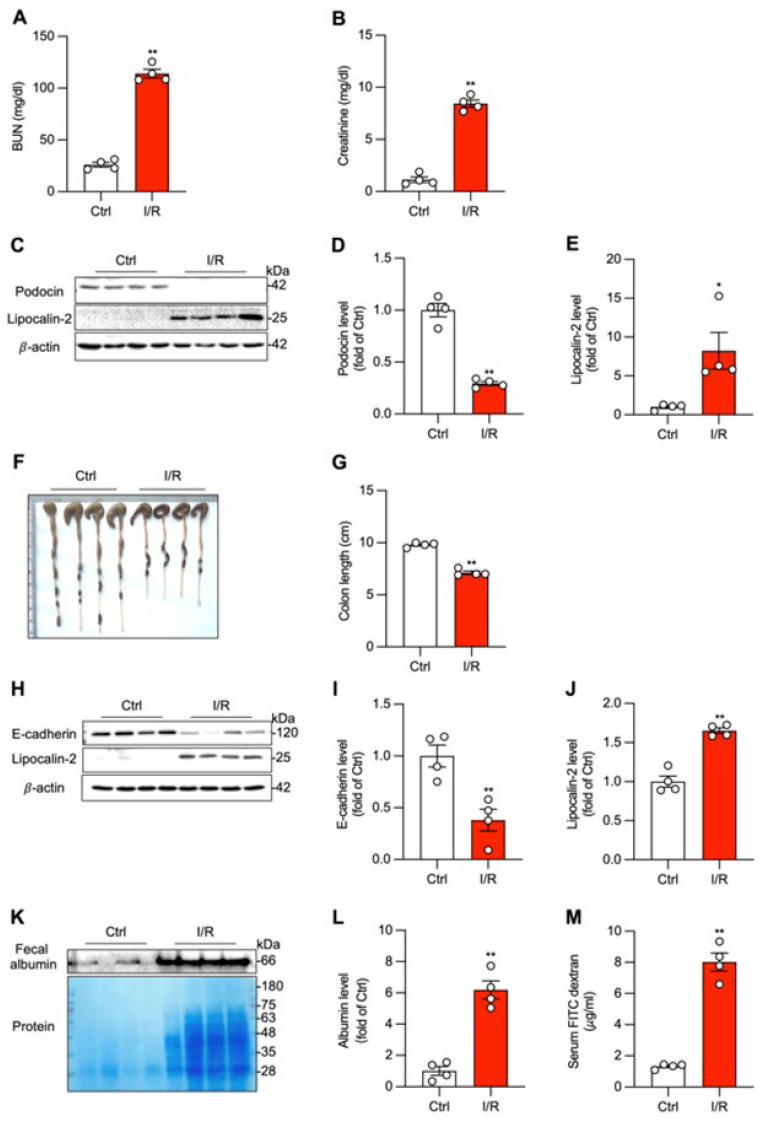
Renal ischemia/reperfusion induces acute kidney injury and remote colonic barrier dysfunction. (**A**,**B**) Renal function was assessed by measuring blood urea nitrogen (BUN) and serum creatinine levels at 24 h after 45 min of bilateral renal pedicle clamping and reperfusion. (**C**–**E**) Renal injury markers were evaluated by Western blot analysis of kidney lysates for podocin and lipocalin-2. Representative blots are shown in (**C**), with quantitative data in (**D**,**E**). (**F**,**G**) Colon length was photographed and measured at 24 h post-I/R. Quantitative results are presented in (**G**). (**H**–**J**) Colonic injury was assessed via determination of protein levels of lipocalin-2 and E-cadherin in colon lysates with Western blot. Quantitative results are presented in (**I**,**J**). (**K**–**M**) Intestinal permeability was assessed via determination of fecal albumin and serum FITC dextran level. Fecal proteins were extracted and assayed for albumin and protein level by Western blot and EZ blue staining (**K**). Quantitative result of fecal albumin is presented in (**L**). Serum from control and I/R mice after gavage administration of FITC dextran for 4 h was collected and assayed for FITC concentration (**M**). Data shown are mean ± SE (*n* = 4; * *p* < 0.05, ** *p* < 0.01 vs. Ctrl). Original images of Western Blotting can be found in Supplementary Materials.

To investigate the pathological mechanisms of organ crosstalk without the confounding effects from the systemic toxicity of the drugs used, a mouse model of renal I/R was used. Following 45 min of bilateral renal artery clamping and 24 h of reperfusion, mice developed severe AKI. This was confirmed by significant elevations in BUN and serum creatinine levels compared with sham-operated controls (Figure 2A,B). The functional decline was paralleled by structural damage to the kidney, including a substantial loss of the glomerular protein podocin and a marked induction of the tubular injury marker lipocalin-2 (Figure 2C–E).

Intriguingly, this localized renal injury caused significant remote pathology in the colon. Grossly, colons from I/R mice were significantly shorter and exhibited signs of hemorrhage compared to sham controls (Figure 2F,G). At the molecular level, this was accompanied by a marked increase in colonic lipocalin-2 expression (Figure 2H,J). To specifically assess intestinal barrier integrity, we examined the adherens junction protein E-cadherin and found its expression to be substantially reduced in the colons of I/R mice (Figure 2H,I). The loss of E-cadherin suggested a compromise in intestinal barrier integrity. This was functionally confirmed by increased intestinal permeability, as demonstrated by a substantial accumulation of total protein and, more specifically, albumin in the feces of I/R mice, indicating leakage from the circulation into the gut lumen (Figure 2K,L). Consistently, a significant flux of orally gavaged FITC-dextran from the gut into the systemic circulation was also observed (Figure 2M). These observations demonstrate that localized kidney injury is sufficient to induce remote intestinal barrier dysfunction.

### 3.3. Gut Microbiota Translocation Exacerbates Renal and Colonic Injury and Increases Mortality

**Figure 3:**
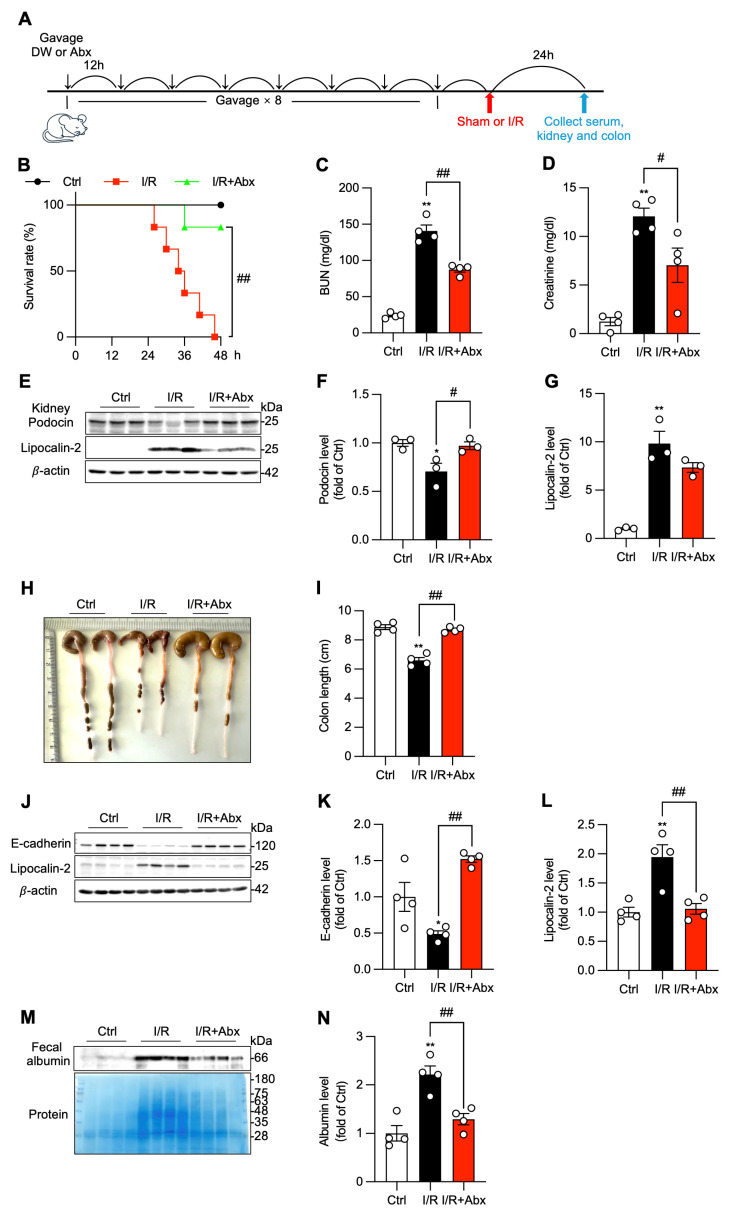
Gut microbiota translocation exacerbates renal and colonic injury and increases mortality. (**A**) Schematic diagram of the experimental design for gut microbiota depletion prior to I/R surgery. Mice were pre-treated with a broad-spectrum antibiotic cocktail (Abx) or vehicle (DW) for 4 days before surgery. (**B**) Effect of Abx treatment on mouse survival rates over 48 h period. Data shown are mean ± SE (*n* = 6, ^##^ *p* < 0.01). (**C**–**G**) Effect of Abx treatment on I/R-induced renal dysfunction. Renal function was assessed via determination of BUN (**C**) and creatinine (**D**) levels (mean ± SE; *n* = 4; ** *p* < 0.01 vs. Ctrl; ^#^ *p* < 0.05, ^##^ *p* < 0.01). Renal injury was evaluated via determination of kidney injury marker podocin and lipocalin-2 (**E**). The densitometric analysis of (**E**) is shown in (**F**,**G**), respectively (mean ± SE; *n* = 3; * *p* < 0.05, ** *p* < 0.01 vs. Ctrl; ^#^ *p* < 0.05). (**H**–**N**) Effect of Abx treatment on I/R-induced changes in colon. Colon length at 24 h after the operation was photographed, measured, and presented as a bar graph in (**H**,**I**). Colonic injury was assessed via determination of protein levels of lipocalin-2 and E-cadherin in colon lysates with Western blot (**J**). Quantitative results are presented in (**K**,**L**). Fecal levels of albumin and proteins were determined via Western blot analysis and EZ blue staining (**M,N**). Data shown are mean ± SE (*n* = 4, * *p* < 0.05, ** *p* < 0.01 vs. Ctrl, ^##^ *p* < 0.01). Original images of Western Blotting can be found in Supplementary Materials.

Based on observations of I/R-induced gut barrier failure, we hypothesized that subsequent translocation of gut microbes or their products contributes to the pathology. To test this, we depleted gut microbiota with a broad-spectrum oral antibiotic cocktail before I/R surgery, using the method we have previously reported [18]. The schematic experimental outline is shown in Figure 3A. Antibiotic pretreatment resulted in a significant improvement in survival over the observation period compared to the untreated I/R group (Figure 3B). This improved survival was associated with significant protection of I/R-induced kidney injury, as shown by the markedly lower serum BUN and creatinine levels (Figure 3C,D) and preservation of glomerular podocin, as well as attenuation of tubular lipocalin-2 expression (Figure 3E–G).

Antibiotic treatment also conferred significant protection to the colon. It preserved colon length (Figure 3H,I), attenuated the I/R-induced downregulation of colonic E-cadherin, and suppressed the expression of colonic lipocalin-2 (Figure 3J–L). Furthermore, the treatment maintained gut barrier integrity, as evidenced by the prevention of albumin leakage into the feces (Figure 3M,N). These results indicate that the translocation of luminal contents following gut barrier dysfunction is a major contributor to both the primary renal injury and the remote colonic damage and is a key factor in I/R-associated mortality.

### 3.4. Antioxidant Treatment Mitigates Renal and Colonic Injury by Restoring Redox Homeostasis

**Figure 4:**
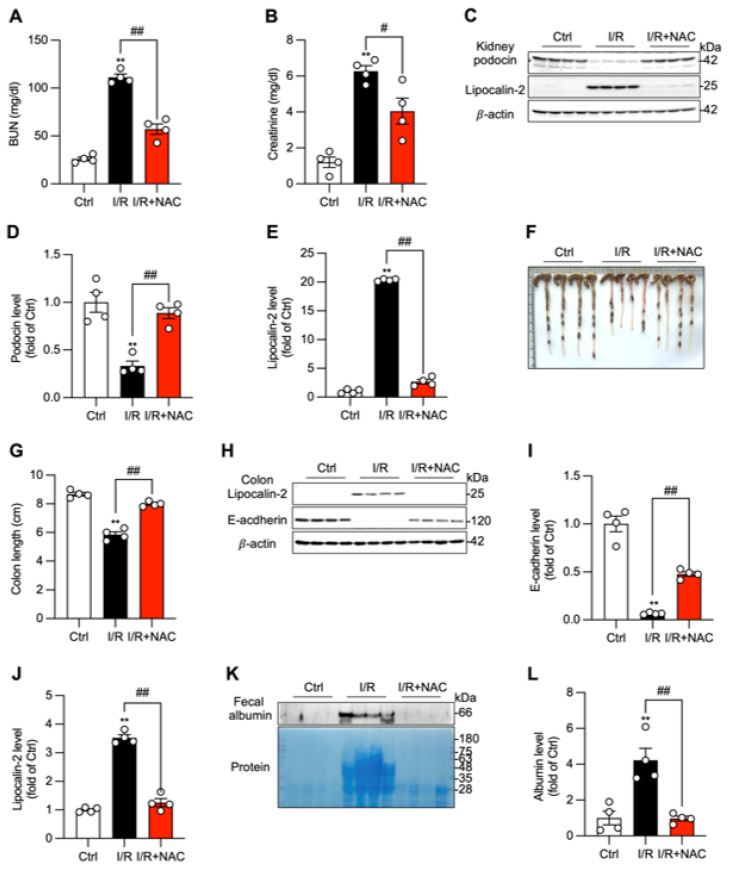
Antioxidant treatment mitigates renal and colonic injury. (**A**,**B**) Effect of NAC treatment on renal function. Mice were treated with NAC (200 mg/kg, twice daily for 3 days). Renal function was assessed via BUN and creatinine levels. (**C**–**E**) Effect of NAC treatment on I/R-induced kidney injury maker podocin and lipocalin-2. (**F**–**L**) Effect of NAC treatment on I/R-induced changes in the colon. Colon lengths at 24 h after the operation were photographed, measured, and presented as a bar graph in (**F**,**G**). Colon injury was determined via assessment of the level of lipocalin-2 and E-cadherin in colon lysates (**H**–**J**), and permeability was assessed via determination of fecal albumin and protein levels via Western blot analysis and EZ blue staining (**K**,**L**). Data shown are mean ± SE (*n* = 4, ** *p* < 0.01 vs. Ctrl, ^#^ *p* < 0.05; ^##^ *p* < 0.01). Original images of Western Blotting can be found in Supplementary Materials.

To investigate the causal role of oxidative stress in this organ crosstalk, mice were treated with the antioxidant NAC before I/R, as we have previously reported [16]. NAC administration significantly attenuated AKI, as demonstrated by lower serum BUN and creatinine levels (Figure 4A,B). This was accompanied by the mitigation of renal structural damage, shown by the preservation of podocin and the reduced induction of lipocalin-2 (Figure 4C–E). NAC treatment also prevented I/R-induced intestinal pathology, preserving colon length, suppressing colonic lipocalin-2 expression, maintaining colonic E-cadherin levels, and controlling the increase in fecal albumin (Figure 4F–L).

**Figure 5:**
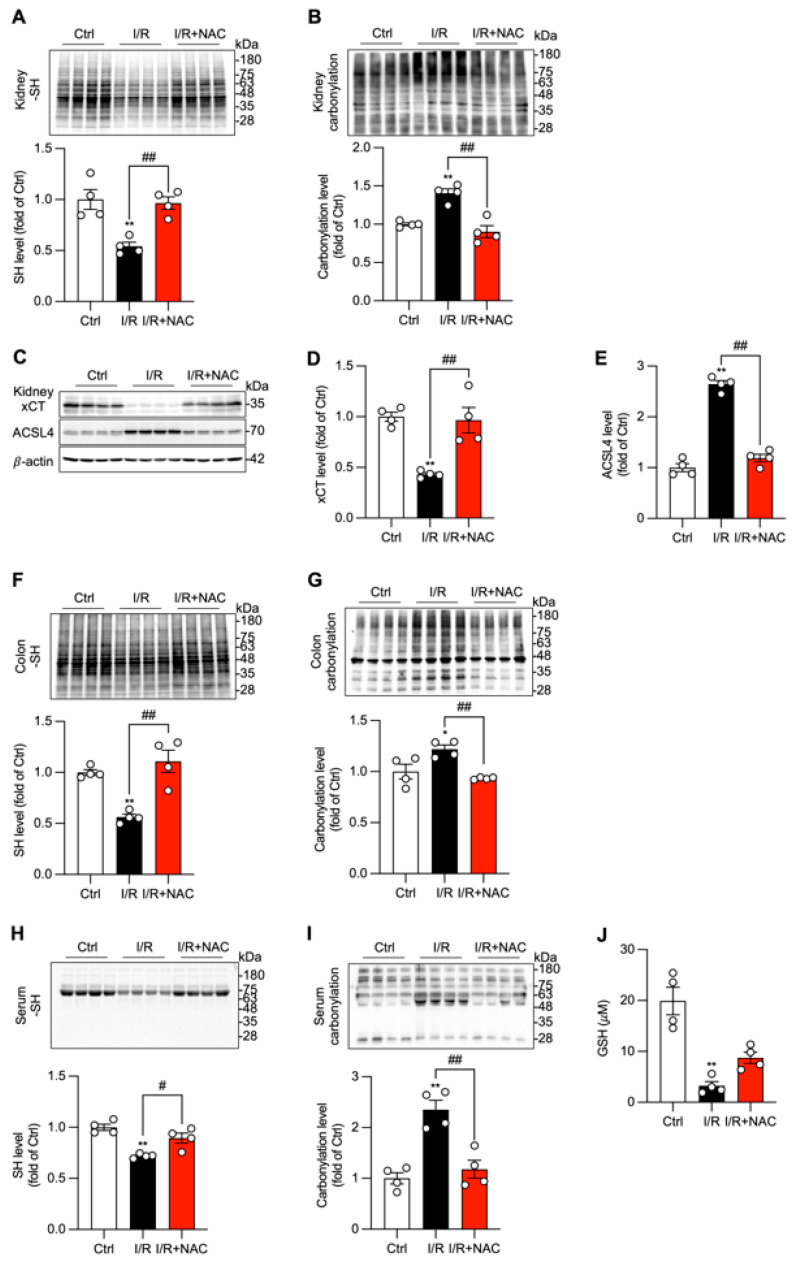
Antioxidant treatment improves local and systemic redox status and kidney ferroptosis. (**A**,**B**) Effects of NAC treatment on the level of -SH groups and carbonylation in kidney tissue. The protein levels of -SH groups and carbonyl formation in kidney lysate were determined by Western blot analysis (upper panel). The densitometric analysis of the blots is shown in the graphs in the lower panel. (**C**–**E**) Effects of NAC treatment on ferroptosis marker xCT and ACSL4 in kidney tissue. (**F**,**G**) Effects of NAC treatment on the level of -SH groups and carbonylation in the colon. (**H**–**J**) Effects of NAC treatment on systemic redox status. Mouse sera from differently treated groups were assayed for the protein level of -SH (**H**) and carbonylation (**I**), as well as GSH (**J**). Data shown are mean ± SE (*n* = 4; * *p* < 0.05, ** *p* < 0.01 vs. Ctrl, ^#^ *p* < 0.05, ^##^ *p* < 0.01). Original images of Western Blotting can be found in Supplementary Materials.

The protective effects of NAC were associated with the restoration of local and systemic redox homeostasis. The I/R-induced depletion of protein-free thiols and the corresponding increase in protein carbonylation were significantly reversed by NAC treatment in the kidney, colon, and serum (Figure 5). Furthermore, NAC suppressed the induction of ferroptosis markers in the kidney, normalizing the expression of xCT and ACSL4 (Figure 5C–E). These findings establish that oxidative stress is a key mechanism driving the pathological kidney–gut interaction and that antioxidant therapy can effectively prevent injury by restoring redox balance and inhibiting downstream cell death pathways.

### 3.5. Serum from I/R Mice Is Cytotoxic and Disrupts Intestinal Epithelial Barrier Function

**Figure 6:**
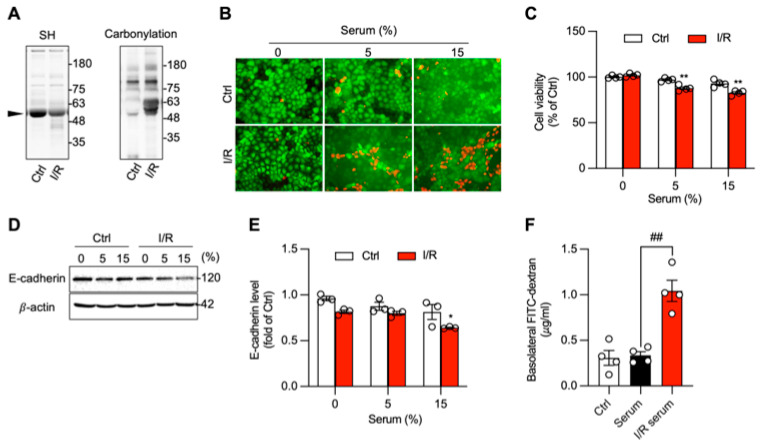
Serum from I/R mice induces intestinal epithelial cell injury and disrupts barrier function. (**A**) Detection of serum -SH and carbonyl level from normal and I/R mice. Note the reduced level of -SH and elevated level of carbonyl at MW around 48~63 kDa (arrowhead). (**B**,**C**) Induction of intestinal epithelial injury by I/R serum. Caco-2 cells were exposed to the indicated concentration of control and I/R serum, and cell viability was determined through Calcein AM/PI staining (**B**) and formazan formation (**C**). Note the elevated number of PI-positive red dead cells in I/R serum-treated cells. (**D**–**F**) Effect of I/R serum on adherent junction protein and cellular permeability. The cellular proteins were extracted and subjected to Western blot analysis for E-cadherin (**D**). The quantitative results of (**D**) are presented in (**E**). The concentration of FITC-dextran across Caco-2 monolayers cultured in the Transwell system, treated with control or I/R serum, was measured (**F**). Data shown are mean ± SE; *n* = 4 for C and F, *n* = 3 for E; * *p* < 0.05, ** *p* < 0.01 vs. Ctrl, ^##^ *p* < 0.01). Original images of Western Blotting can be found in Supplementary Materials.

The systemic nature of the oxidative stress suggested the involvement of circulating pathogenic factors. Analysis of serum from I/R mice revealed a state of oxidative modification, with decreased protein thiol levels and increased protein carbonylation, particularly at a molecular weight of 48~66 kDa (Figure 6A, arrowhead). To test its biological activity, this serum was applied to cultured Caco-2 intestinal epithelial cells. Compared with control-mouse serum, I/R serum induced significant cell death, as shown by an increase in PI-positive cells, and reduced overall cell viability, as measured by a WST assay (Figure 6B,C).

This cytotoxic effect was associated with a disruption of epithelial barrier function. Treatment with I/R serum led to a significant reduction in E-cadherin expression (Figure 6D,E) and a corresponding increase in the permeability of the Caco-2 cell monolayer to FITC-dextran (Figure 6F).

**Figure 7:**
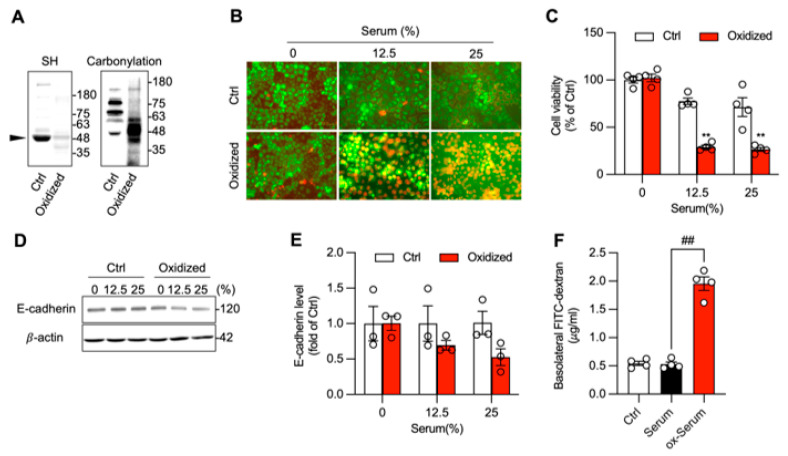
Exogenously oxidized serum proteins induce intestinal epithelial cell death and disrupt cellular barrier function. (**A**) Characterization of exogenously oxidized serum proteins. Sera from normal control mice were either oxidized with vitamin C plus copper or left untreated. After removing small molecules by dialysis, the proteins were collected and characterized for protein oxidation by assessing-SH and carbonyl levels. Note the decreased -SH and increased carbonyl formation in protein at MW around 48~63 kDa (arrowhead). (**B**,**C**) Induction of intestinal epithelial injury by exogenously oxidized serum proteins. Caco-2 cells were exposed to the indicated concentration of control and oxidized serum protein. Cell viability was determined through Calcein AM/PI staining (**B**) and formazan formation (**C**). Note the elevated number of PI-positive red dead cells in the cells treated with exogenously oxidized serum proteins. (**D**–**F**) Effect of exogenously oxidized protein on adherent junction and cellular permeability. Cells were treated with control or exogenously oxidized serum proteins for 6 h and assayed for E-cadherin levels (**D**). The quantitative results of (**D**) are presented in (**E**). The cellular barrier integrity was assessed via the evaluation of the amount of FITC-dextran passing through Caco-2 monolayers cultured in the Transwell system after treatment with control or oxidized serum protein for 24 h (**F**). Data shown are mean ± SE (*n* = 4 for C and F, *n* = 3 for E; ** *p* < 0.01 vs. Ctrl, ^##^ *p* < 0.01). Original images of Western Blotting can be found in Supplementary Materials.

To determine whether oxidative modification of serum proteins was sufficient to cause this pathology, serum from normal mice was artificially oxidized in vitro using chemicals that initiate a Fenton reaction [16,27]. This ox-Serum exhibited an oxidative profile like I/R serum proteins, displaying a reduced level of thiols and an increased carbonylation, especially at the location around 48~66 kDa (Figure 7A, arrowhead). When applied to Caco-2 cells, ox-serum recapitulated the effects of I/R serum, inducing cell death (Figure 7B), reducing cell viability (Figure 7C), decreasing E-cadherin, and increasing monolayer permeability (Figure 7D–F). These results indicate that oxidized serum proteins are sufficient to induce intestinal epithelial cell injury and barrier dysfunction.

### 3.6. Oxidized Albumin Is Sufficient to Induce Intestinal Epithelial Injury

**Figure 8:**
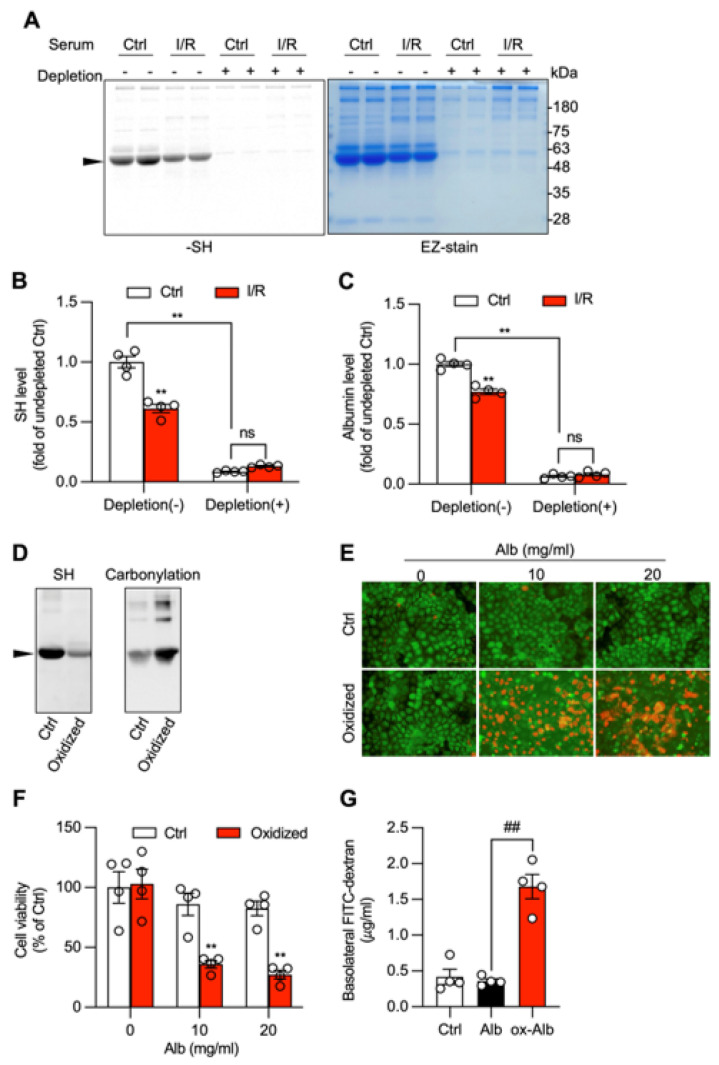
Oxidized albumin is sufficient to induce intestinal epithelial injury. (**A**–**C**) Identification of albumin as the predominant protein to be oxidized in I/R serum. Sera from control and I/R mice were treated either with albumin-depleting reagent (depletion +) to remove albumin or left untouched (depletion −). The processed sera were assayed for -SH level with maleimide-labelling assay. Note the reduced -SH groups in untreated I/R sera as compared to control at MW around 48~63, and their disappearance after albumin depletion (arrowhead). The changes in serum -SH and albumin before and after albumin depletion are shown in (**B**) and (**C**), respectively. (**D**) Characterization of oxidized albumin. Albumin was oxidized with vitamin C plus copper and confirmed for its oxidation via assessment of -SH and carbonyl level. Note the decreased -SH and increased carbonyl formation after oxidation (arrowhead). (**E**,**F**) Induction of intestinal epithelial injury by oxidized albumin. Caco-2 cells were exposed to the indicated concentration of control and oxidized albumin. Cell viability was determined through Calcein AM/PI staining (**E**) and formazan formation (**F**). Note the elevated number of PI-positive red dead cells in the cells treated with exogenously oxidized albumin. (**G**) Effect of ox-Alb on cellular permeability. Cells were treated with control or ox-Alb (20 mg/mL), and assayed for barrier integrity via the evaluation of the amount of FITC-dextran passing through Caco-2 monolayers cultured in the Transwell system. Data shown are mean ± SE (*n* = 4; ** *p* < 0.01 vs. Ctrl, ^##^ *p* < 0.01, ns = non-significant). Original images of Western Blotting can be found in Supplementary Materials.

Given that albumin is the most abundant serum protein, it should be the main protein oxidized in I/R serum and is most likely the leading candidate in mediating the toxic effect. Consistent with this notion, depleting albumin from I/R serum removed the major protein band detected by Coomassie stain and led to a significant reduction in total protein thiol content, confirming albumin as the major carrier of free thiols and the primary oxidized serum protein (Figure 8A–C).

To test whether oxidative modification of albumin is sufficient to confer pathogenic activity, bovine serum albumin (BSA) was oxidized in vitro using the method described above (ox-Alb) and previously reported [16,27]. This oxidative treatment resulted in a pronounced reduction in thiol level, but a marked increase in carbonylation, similar to the state of ox-Alb observed in vivo (Figure 8D). When applied to Caco-2 cells, ox-BSA induced cell death and reduced cell viability (Figure 8E,F). Furthermore, ox-BSA alone was sufficient to disrupt epithelial barrier function, leading to a significant increase in FITC-dextran permeability across the Caco-2 monolayer (Figure 8G). These findings demonstrate that the oxidatively modified albumin is sufficient to induce intestinal epithelial cell injury and barrier failure. It could be a potentially important circulating mediator in the pathological crosstalk between the kidney and the gut.

## 4. Discussion

In this study, we characterized systemic oxidative stress and oxidized serum proteins as critical mediators of pathological inter-organ crosstalk. We demonstrated that renal I/R injury triggers intestinal barrier dysfunction, facilitating the translocation of gut microbiota into the circulation. This microbial invasion acts as a potent amplifier, exacerbating the primary renal injury and driving mortality. Furthermore, we established that this vicious, reciprocal interaction is orchestrated by systemic oxidative stress. Specifically, we characterized oxidized serum albumin not merely as a biomarker, but as a pathogenic effector that directly compromises intestinal epithelial integrity. These findings suggest that targeting oxidative stress and oxidized serum proteins could be an effective way to disrupt progression toward MODS.

Our study expands the current understanding of the gut–kidney axis. Consistent with clinical observations and experimental data [30,31], we confirmed that primary kidney injury induced remote intestinal pathology. To dissect the underlying mechanism, we utilized a renal I/R model. This approach offers a distinct methodological advantage over chemical induction models, such as APAP and Dox, where it is often difficult to distinguish the drug’s direct systemic toxicity from the secondary consequences of organ failure. By employing a localized ischemic insult, we demonstrated that signals originating solely from the injured kidney are sufficient to trigger colonic injury, manifested by reduced E-cadherin expression, increased permeability, and elevated injury markers. These results align with previous reports linking AKI and CKD to gut barrier failure and subsequent sepsis [30,32], confirming that the kidney-to-gut pathway is a fundamental aspect of the pathology.

A crucial finding of this work is the characterization of the gut as the main factor causing systemic mortality. We hypothesized that barrier dysfunction allows for the invasion of gut microbiota, which subsequently worsens the primary injury. By depleting the gut microbiota with a broad-spectrum antibiotic cocktail before renal I/R, we provided strong evidence that the microbiota acts as a pathological amplifier. Antibiotic treatment significantly attenuated renal injury and improved survival. This supports the concept that when the intestinal barrier fails, microbes and endotoxins translocate and exaggerate the initial inflammation, oxidative stress, and tissue injury. These observations align well with our previous work, which showed that the invasion of gut bacteria exacerbated TNBS-induced colitis and kidney injury via mechanisms involving oxidative stress induction [18]. Other studies in sepsis and limb ischemia also support the notion that the gut is the pivotal organ behind MODS [33,34].

A central aim of this study was to identify the mediators and mechanisms that transmit injury signals from the kidney to the gut. Our group has recently reported that oxidative stress is a common pathogenic mechanism across various organ diseases, including colitis, Dox-induced cardiotoxicity, and APAP-induced hepatotoxicity [18,19,20,27,35]. Furthermore, gut injury induced by diverse local and systemic insults—such as TNBS or DSS colitis and sleep deprivation—can be effectively prevented by antioxidants [18,35,36]. Based on this evidence, it is reasonable to hypothesize that oxidative stress plays a critical role in the transmission of remote organ injury to the gut. Consistent with this hypothesis, we observed that antioxidant therapy completely prevented both renal and colonic injury in the current model.

However, the specific molecular mechanism by which damaging signals are transmitted between organs remains to be fully elucidated. Because reactive oxygen species (ROS), such as superoxide, are highly reactive and short-lived, they are unlikely to function as direct, long-distance signaling mediators. This limitation suggests the existence of a stable, circulating carrier that can convey oxidative stress. In support of this concept, we found that serum collected from renal I/R mice induced barrier dysfunction and significant cytotoxicity in cultured intestinal epithelial cells. Intriguingly, these effects were recapitulated by exogenously oxidized serum. Since this experimental preparation excludes the influence of cellular components, inflammatory mediators, and uremic toxins generated during I/R, the results implicate oxidized proteins in the induction of cytotoxicity. As albumin is the predominant plasma protein susceptible to oxidation, and purified ox-Alb exerted direct cytotoxic effects on epithelial cells, we conclude that ox-Alb is the principal mediator responsible for transmitting the damaging signal from the kidney to the gut.

A critical question remains: how does circulating albumin interact with intestinal epithelial cells when they are spatially separated by the vascular endothelium under physiological conditions? We propose that this interaction occurs through a sequential compromise of tissue barriers. Systemic oxidative stress and inflammation associated with AKI are known to increase vascular permeability [37,38]. This endothelial dysfunction likely enables ox-Alb to extravasate into the interstitial space, thereby gaining access to the basolateral surface of the intestinal epithelium. Consistent with this hypothesis, we observed hemorrhagic lesions in the colons of I/R mice. Furthermore, albumin levels were markedly increased in feces. Together, these observations suggest that ox-Alb not only reaches the basolateral surface but also traverses the epithelium, potentially contacting the apical surface.

Once situated within the tissue microenvironment adjacent to intestinal epithelial cells, ox-Alb may exert toxicity through multiple mechanisms. First, intestinal epithelial cells express scavenger receptors (e.g., CD36) and RAGE, which bind modified proteins [39,40]. The endocytosis of ox-Alb via these pathways can disrupt intracellular redox homeostasis, activate redox signaling, interfere with mitochondrial and proteasome functions [41,42,43], or induce ferroptosis, as we recently reported in renal tubular cells [27]. Second, albumin functions as a carrier for uremic toxins (e.g., indoxyl sulfate) and microbial products (e.g., LPS) [44,45]. The local release of these toxic payloads may indirectly damage cells. Importantly, although we did not directly assess the in vivo effects of ox-Alb on intestinal permeability in this study, the recent literature provides strong evidence that the direct in vivo administration of ox-Alb is sufficient to induce intestinal epithelial barrier loss [26,40,46]. These findings reinforce our conclusion that ox-Alb is a critical mediator that transmits injury signals from the kidney to the gut by disrupting intestinal barrier function.

It is important to note that previous studies have implicated inflammatory mediators (TNF-α, IL-6), activated inflammatory cells, and uremic toxins in the transmission of inter-organ injury [47,48,49,50]. In the context, ox-Alb is likely not the only factor involved in inter-organ injury; rather, it probably works in synergy with other mediators. For instance, cytokines may increase vascular permeability, facilitating ox-Alb accumulation. In contrast, the accumulated ox-Alb alters redox status and activates pro-inflammatory signaling pathways [22,51,52], thereby exaggerating local inflammation, oxidation, and injury. More detailed studies are needed to understand the precise interplay among these different factors and pathways.

Our findings could have significant implications. First, our study reframes the pathological role of albumin in kidney disease. Beyond its role as a biomarker or a promoter of renal diseases via its actions on tubular cells after reabsorption [22], we identify ox-Alb as a systemic mediator that transmits damaging signals and initiates a vicious gut–kidney cycle. Second, although this study focused on the kidney–gut axis, these ox-Alb-mediated mechanisms may also operate in other critical situations in which oxidative stress plays a central role, such as acute liver failure, severe pancreatitis, and sepsis [53]. Third, for decades, antioxidant therapies have shown promise in preclinical models but have largely failed in clinical trials [54]. Our work suggests that, in addition to focusing on scavenging transient ROS, strategies against the stable, long-lived ox-Alb molecule should be developed to disrupt the vicious crosstalk between the gut and organs. These strategies could involve preventing ox-Alb formation, enhancing its clearance, and/or reducing ox-Alb back to its reductive form.

Of note, our study has limitations. While we demonstrated the cytotoxicity of ox-Alb in vivo and the impact of serum transfer, the precise molecular signaling pathways downstream of ox-Alb uptake in intestinal cells warrant deeper investigation. In addition, our findings of a strong correlation between ox-Alb levels and gut barrier dysfunction in the mouse model need to be validated in clinical settings. Furthermore, the study was performed exclusively in male mice; it remains to be validated whether the finding could also be similarly achieved in and apply to female subjects.

## 5. Conclusions

In conclusion, we characterize systemic oxidative stress and the resulting generation of ox-Alb as central mechanisms in inter-organ signaling and ox-Alb functions as a stable pathogenic mediator that transmits injury from the kidney to the gut, compromising barrier integrity. The resulting microbial translocation establishes a pathological feedback loop that amplifies systemic injury and mortality. By shifting the focus from transient ROS to the stable, modified proteins they generate, our work provides a novel mechanistic insight into understanding MODS. It identifies ox-Alb as a promising therapeutic target.

## Data Availability

The original contributions presented in this study are included in the article/Supplementary Material. Further inquiries can be directed to the corresponding author.

## Supplementary Materials

The following supporting information can be downloaded at https://www.mdpi.com/article/10.3390/biom16030462/s1. Original images of Western Blotting can be found in Supplementary Materials.

## Abbreviations

The following abbreviations are used in this manuscript:

I/R	Ischemia/reperfusion
CKD	Chronic kidney disease
SIRS	Systemic inflammatory response syndrome
MODS	Multiple organ dysfunction syndrome
ROS	Reactive oxygen species
NOX	NADPH oxidases
Cys34	Cysteine-34
ox-Alb	Oxidized albumin
AKI	Acute kidney injury
PI	Propidium Iodide
GSH	Glutathione
DSS	Sodium dextran sulfate
Dox	Doxorubicin
TNBS	Trinitrobenzenesulfonic acid
BUN	Blood urea nitrogen
DTT	Dithiothreitol
AA	Ascorbic acid
Alb-SSH	Sulfhydrylated albumin
H_2_O_2_	Hydrogen Peroxide
NAC	N-acetylcysteine
NaHS	Sodium hydrosulfide hydrate
IP	Intraperitoneal injection
-SH	Sulfhydryl
SSA	Sulfosalicylic acid
SE	Standard error
BSA	Bovine serum albumin
