# Supplementary material for: Systemic Oxidative Stress and Oxidized Albumin Mediate the Pathogenic Kidney-to-Gut Crosstalk by Disrupting Intestinal Barrier Integrity
Source: Biomolecules. 2026 Mar 18;16(3):462. doi: 10.3390/biom16030462 (PMC13024482; doi:10.3390/biom16030462)

Fig 1A Colon length

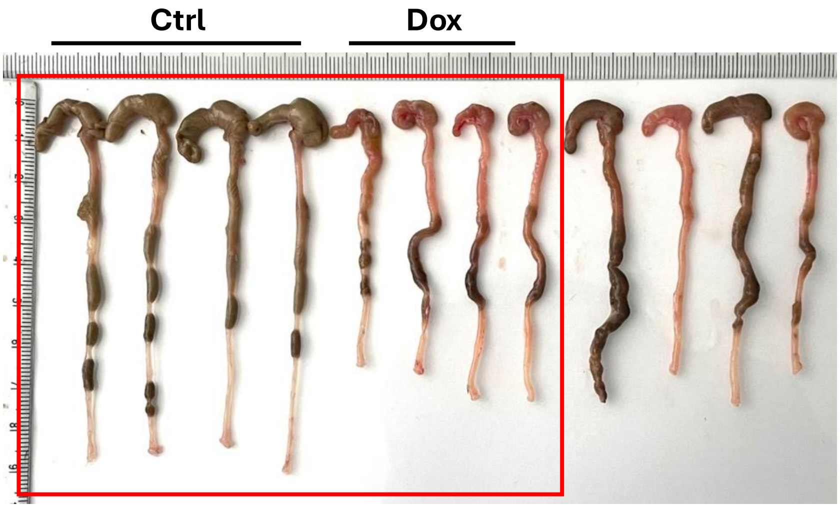

Fig 1C Lipocalin-2

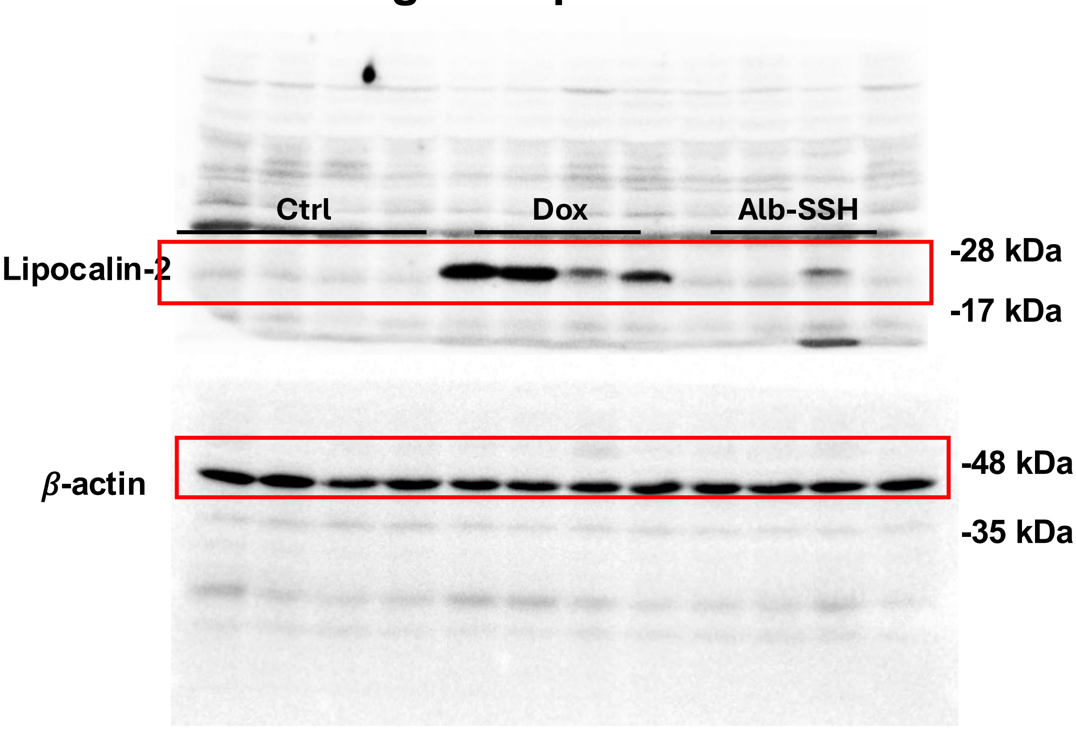

Fig 1E Serum -SH

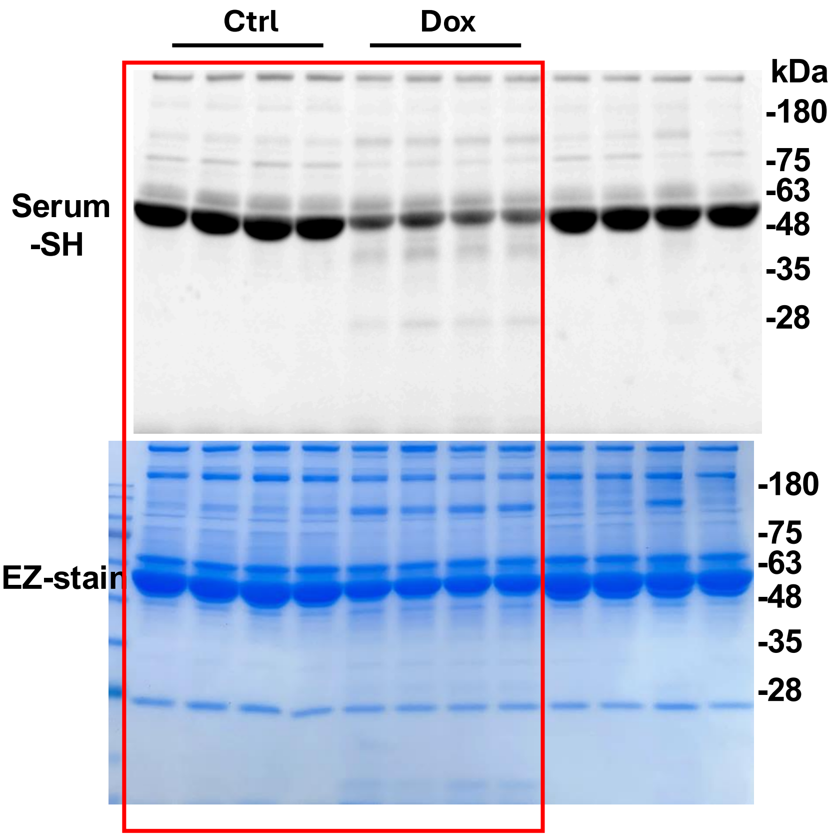

Fig 1G Colon length

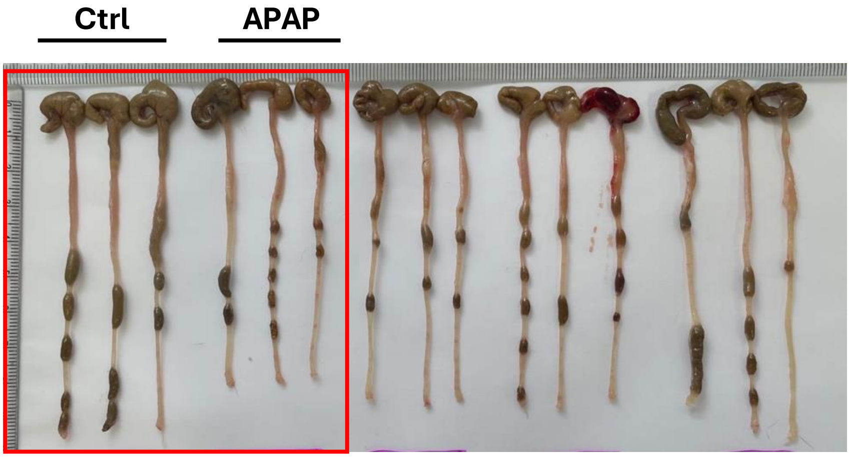

Fig 1I Cleved caspase-3

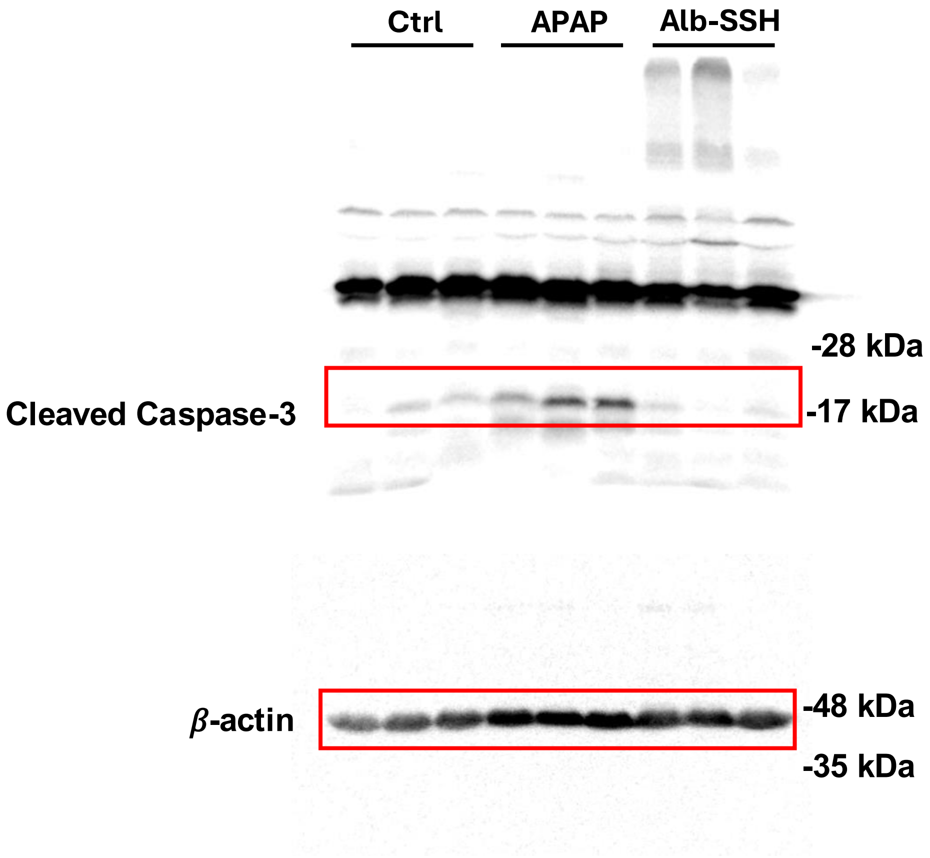

Fig 1K Serum -SH

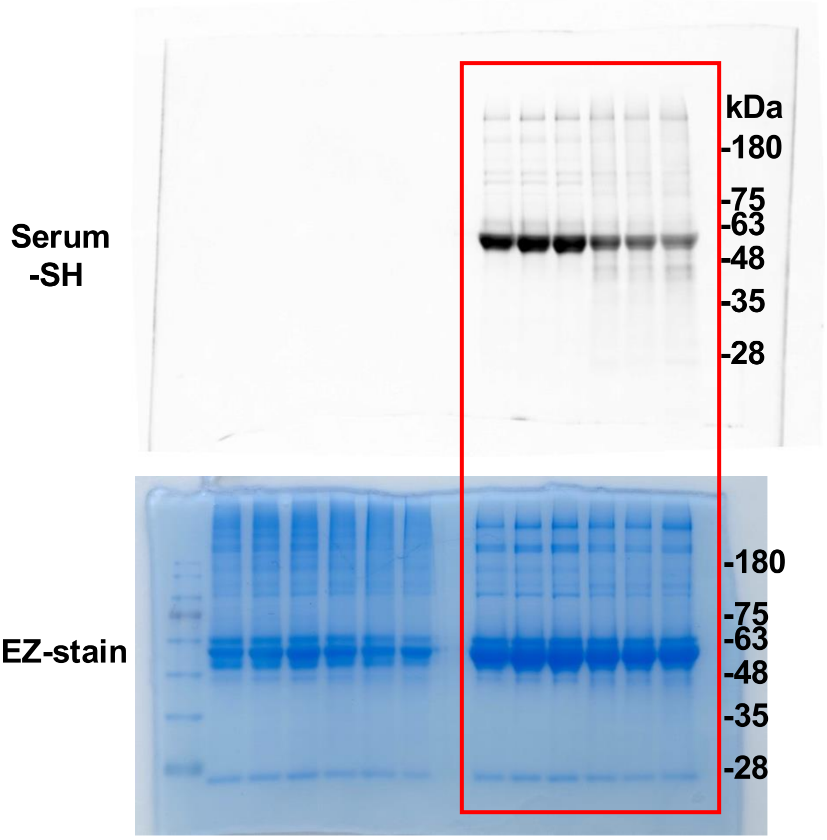

Fig 2C Kidney podocin & lipocalin-2

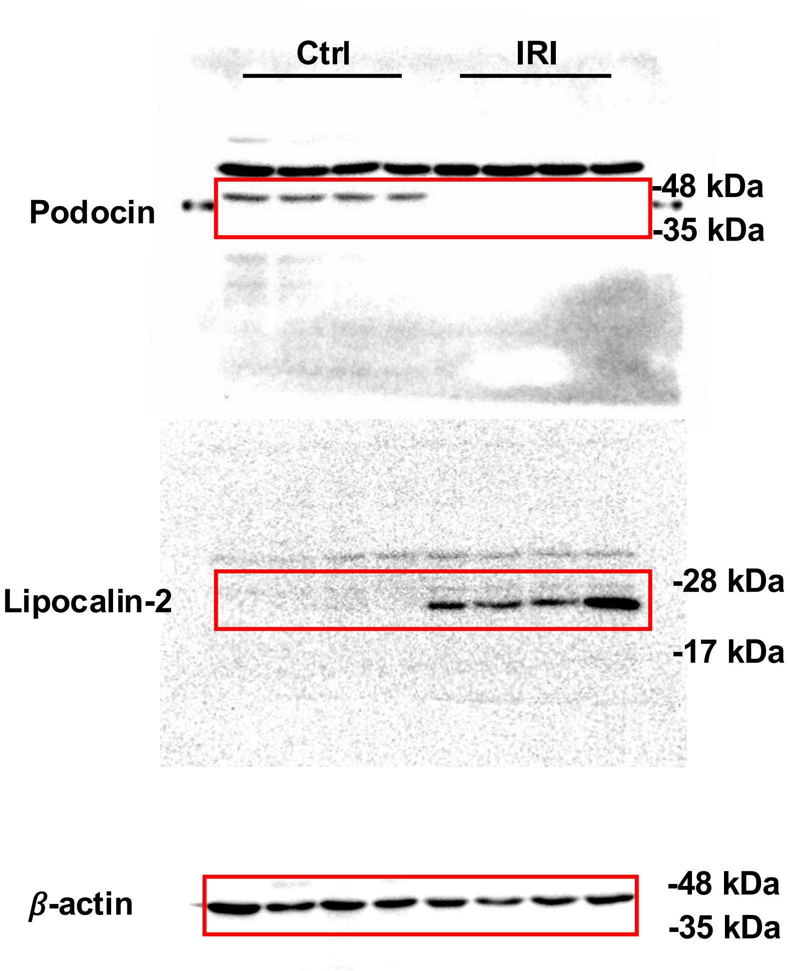

Fig 2F Colon length

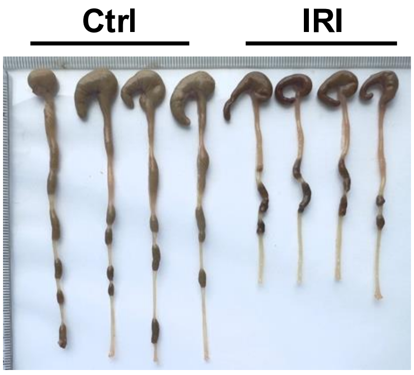

Fig 2H Colon E-cadherin & lipocalin-2

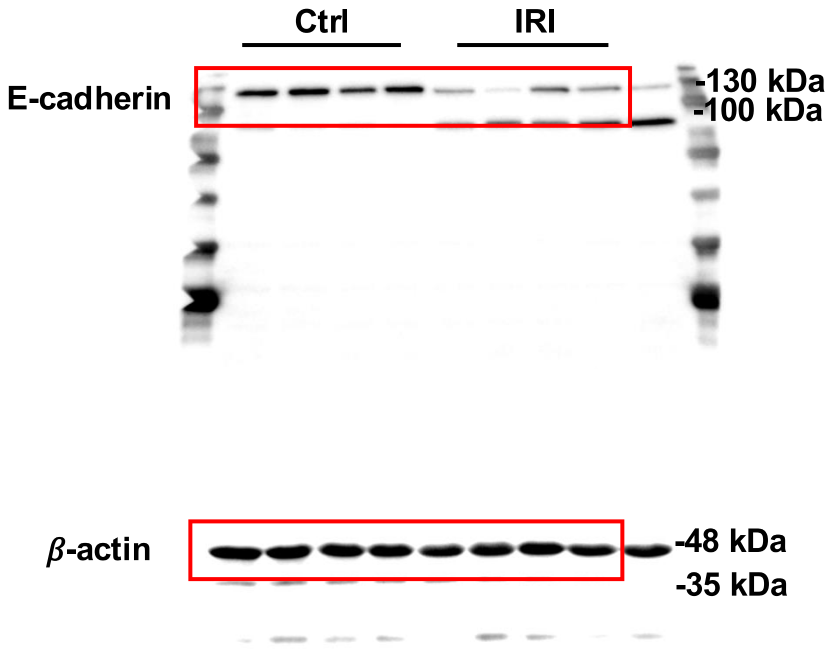

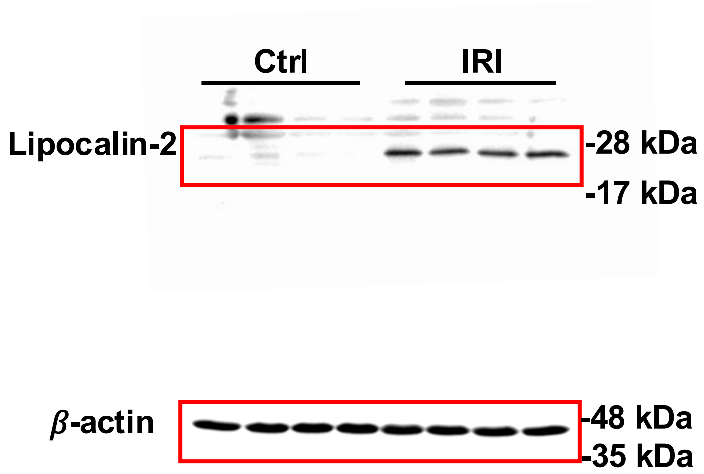

Fig 2K Fecal albumin

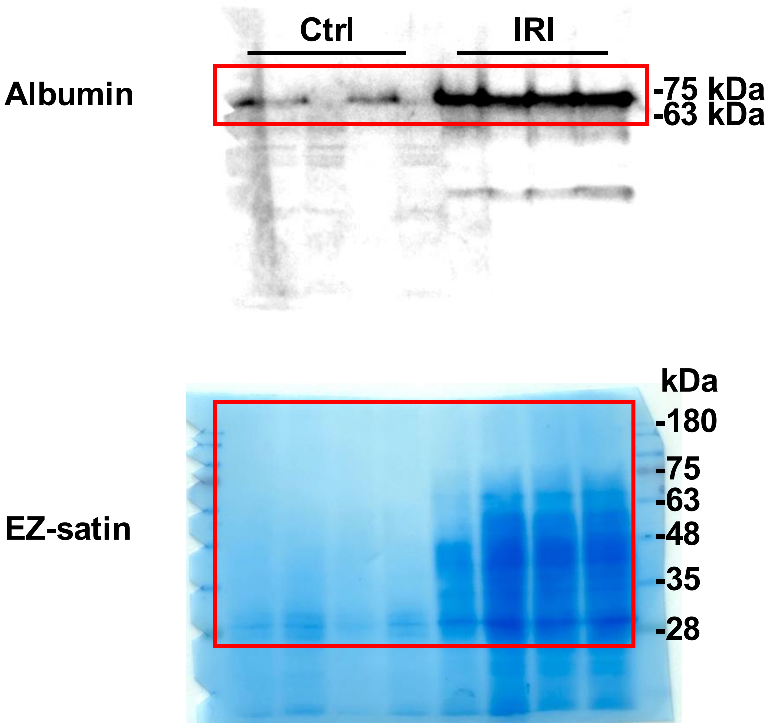

Fig 3E Kidney podocin & lipocalin-2

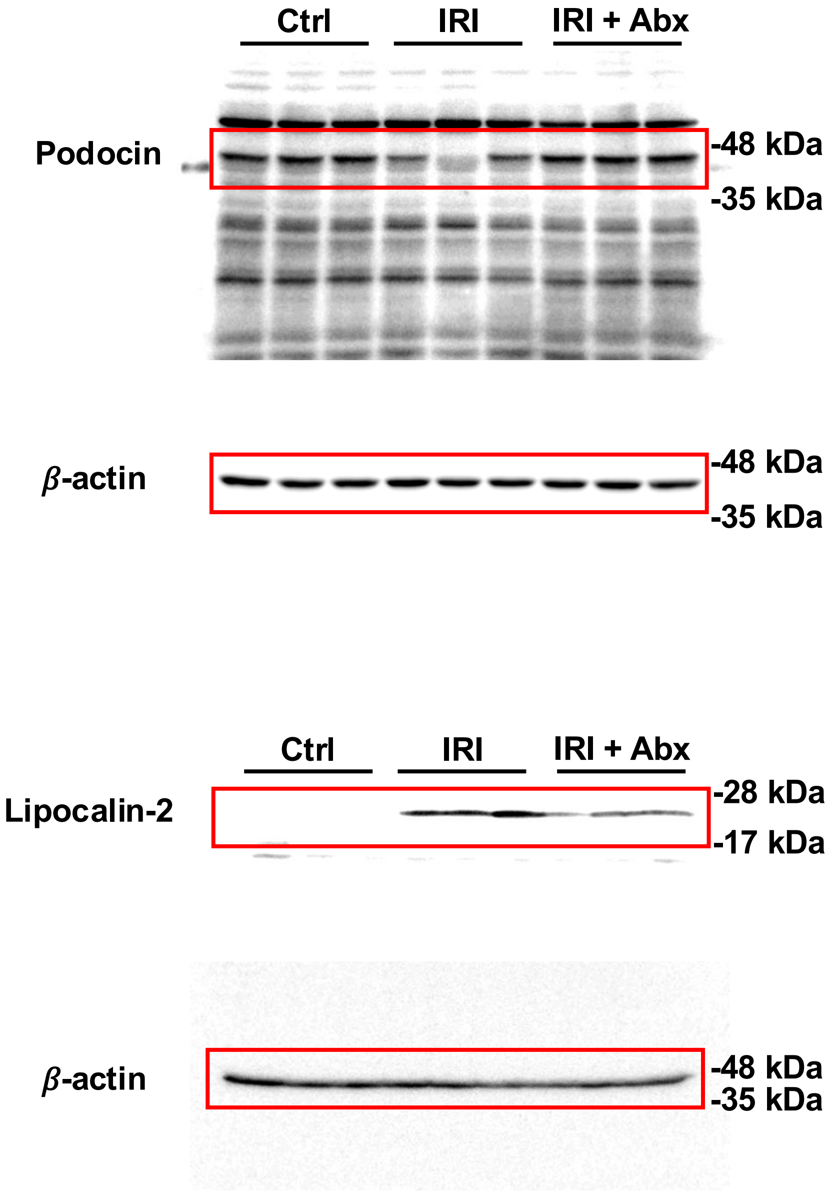

Fig 3H Colon length

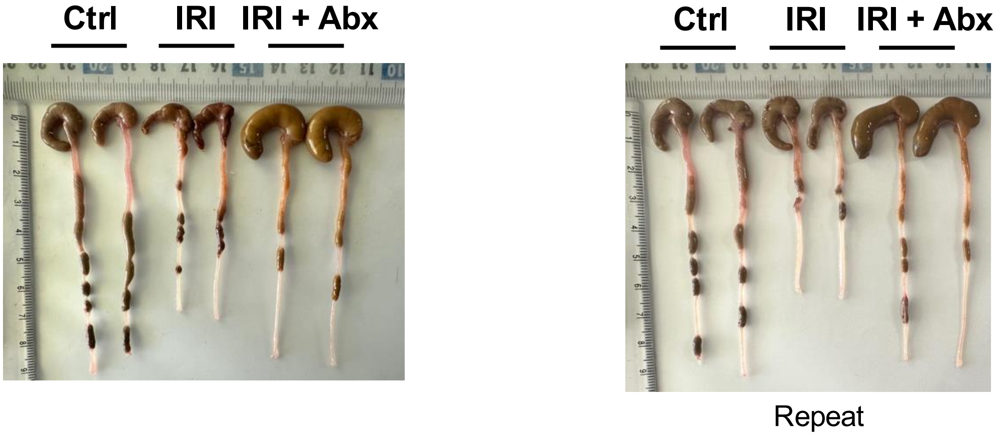

Fig 3J Colon E-cadherin & lipocalin-2

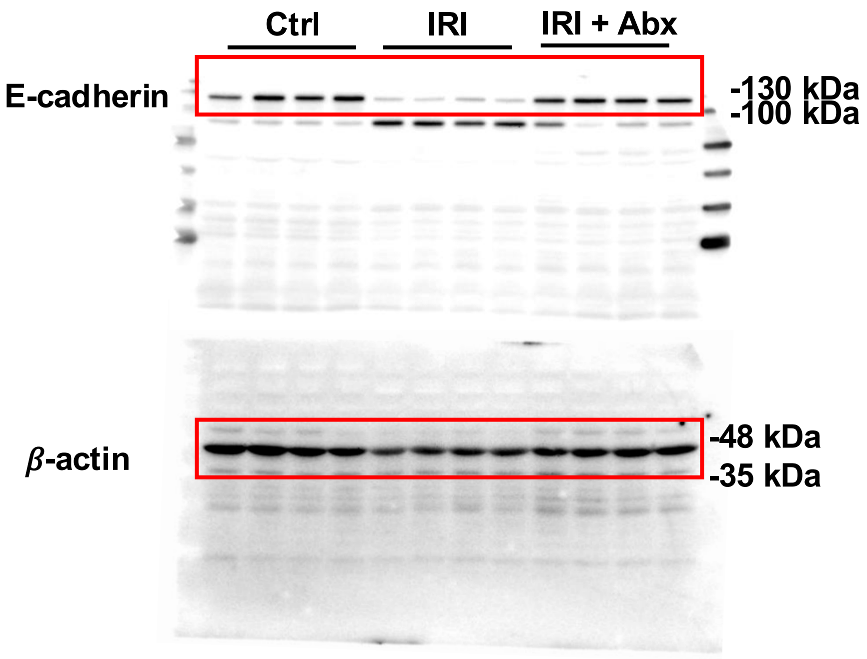

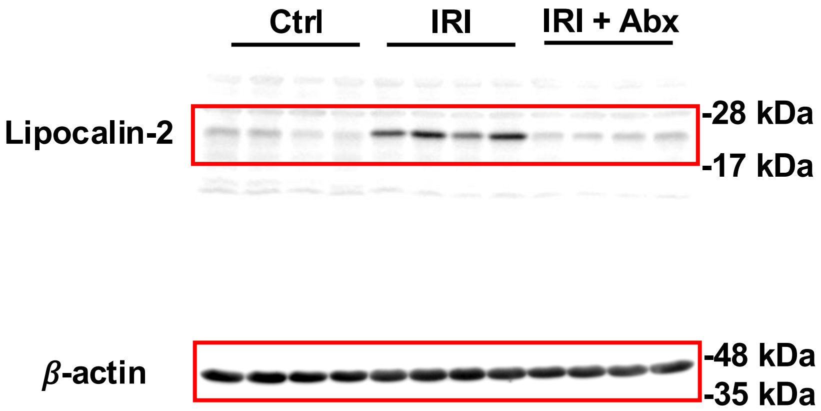

Fig 3M Fecal albumin

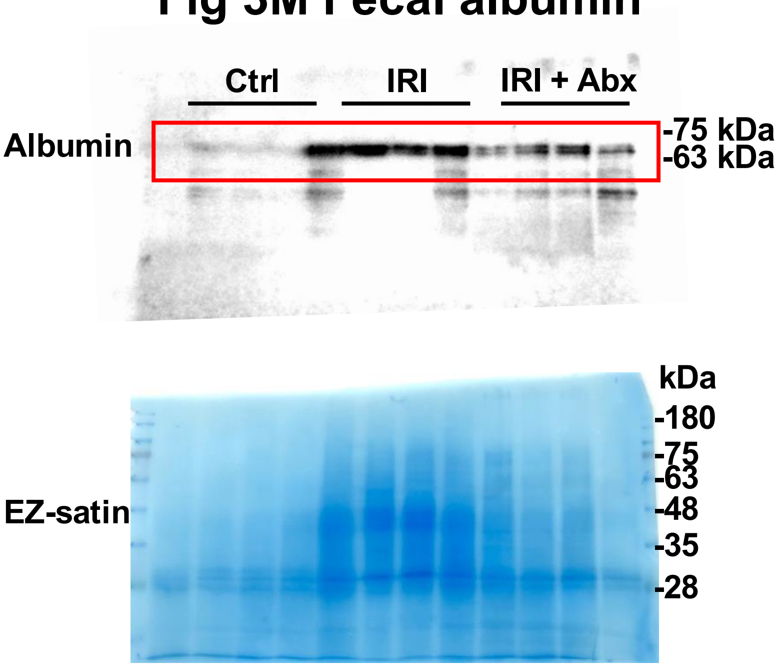

Fig 4C Kicney podocin & lipocalin-2

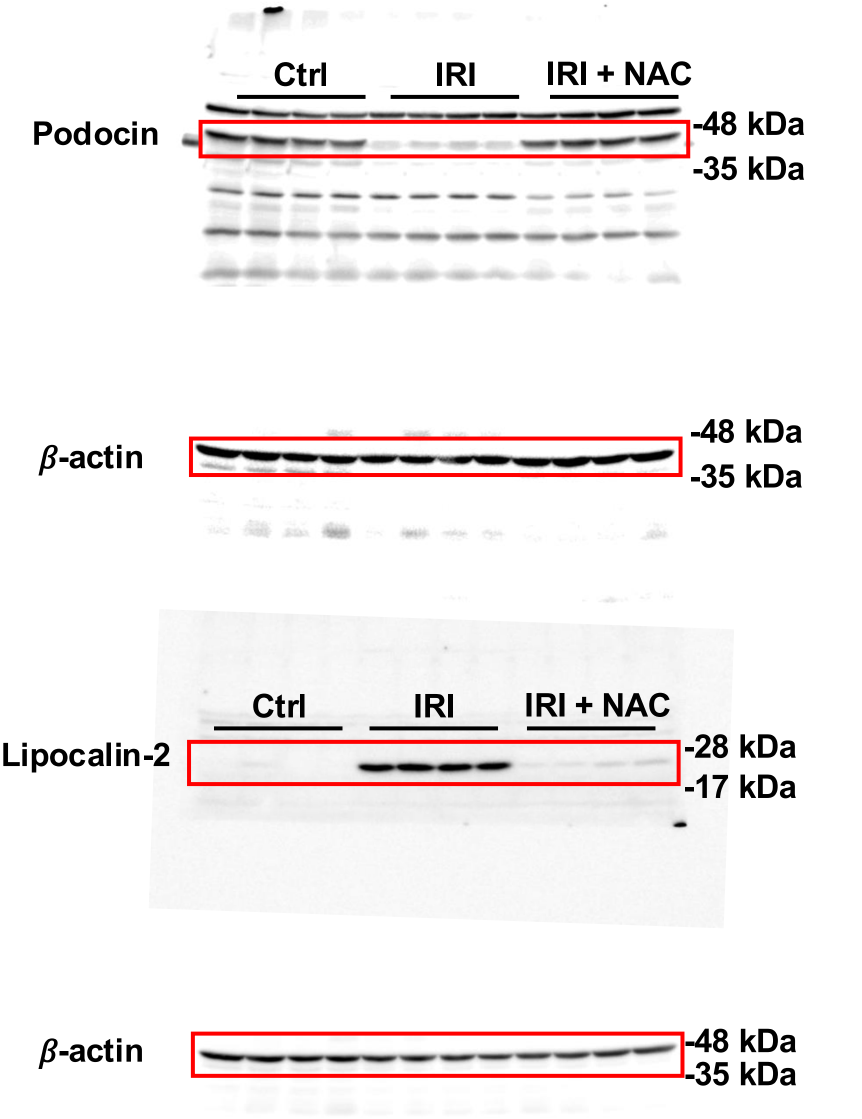

Fig 4F Colon length

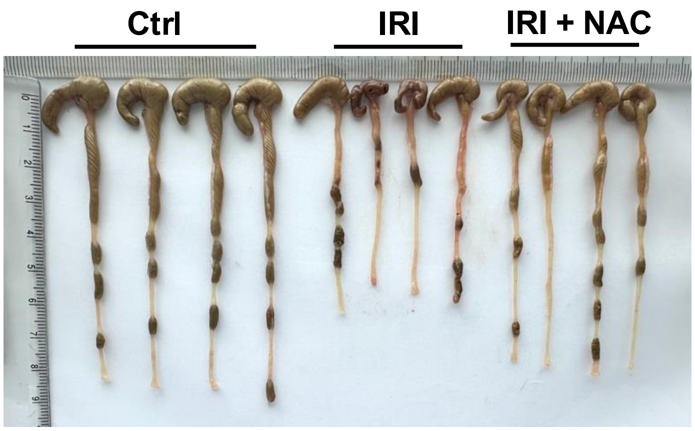

Fig 4H Colon E-cadherin & lipocalin-2

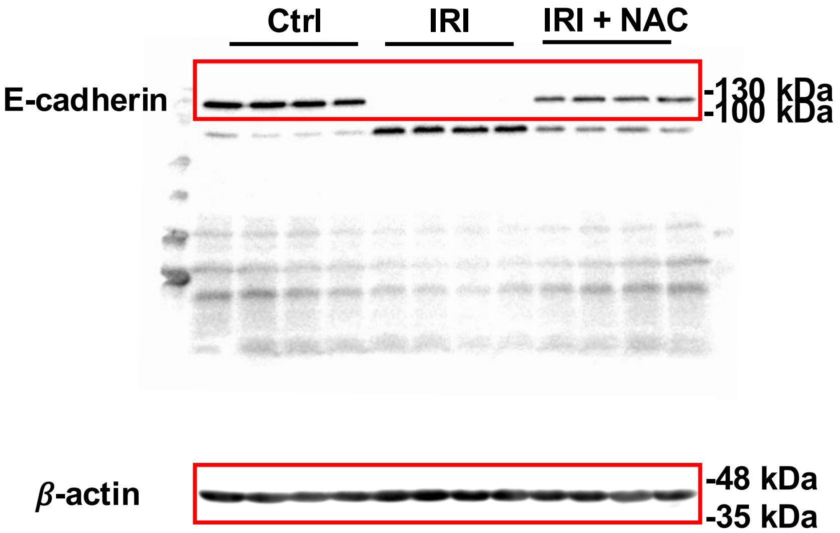

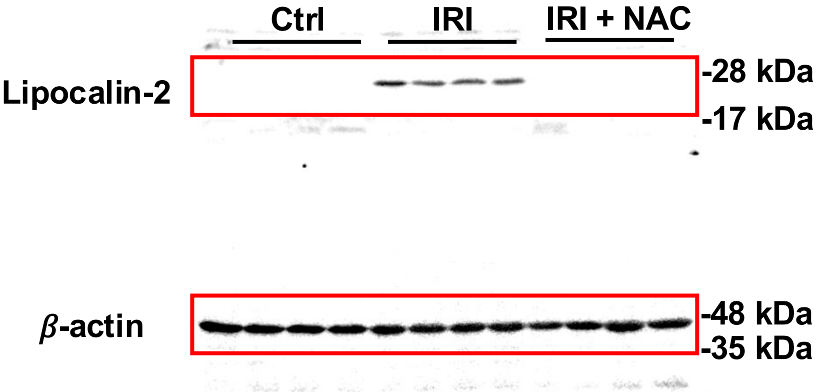

Fig 4K Fecal albumin

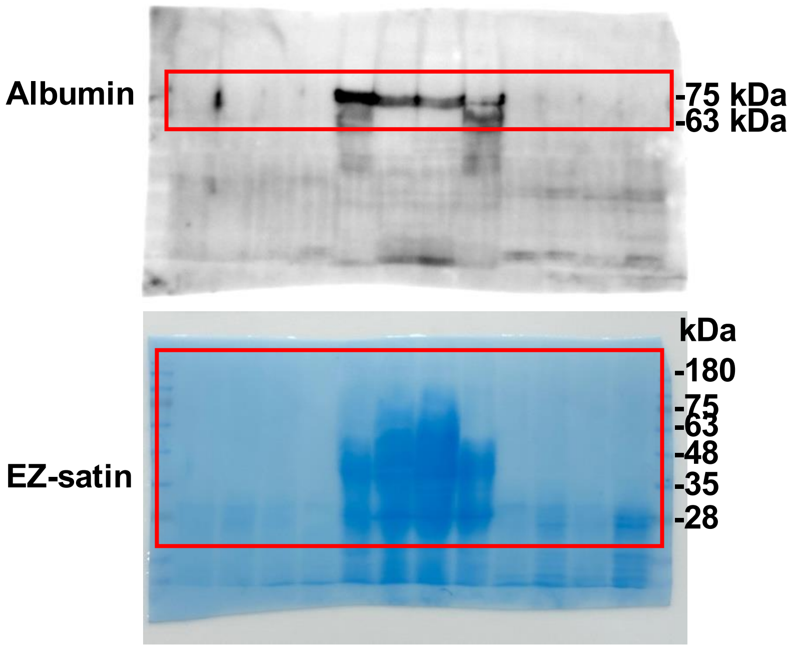

Fig 5A Kidney -SH

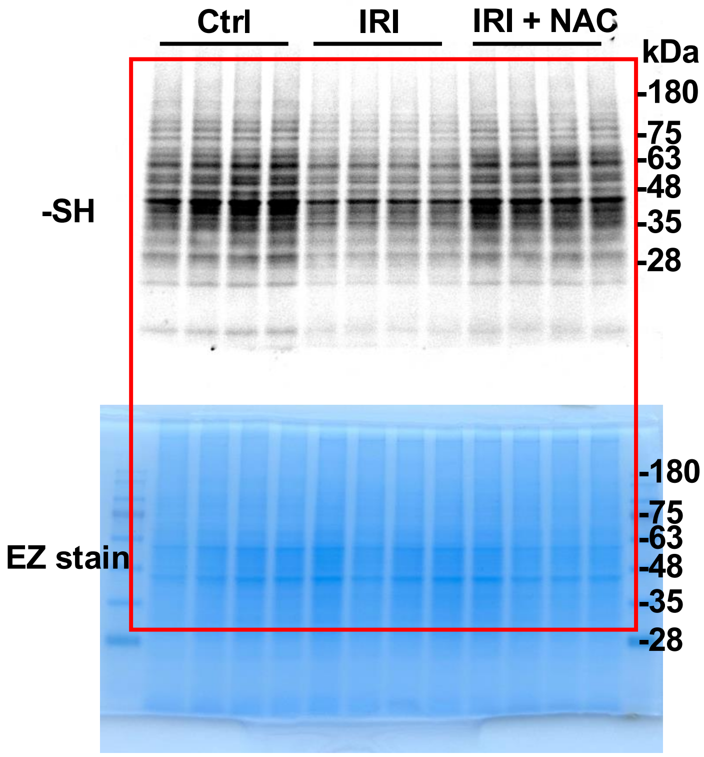

Fig 5B Kidney -carbonylation

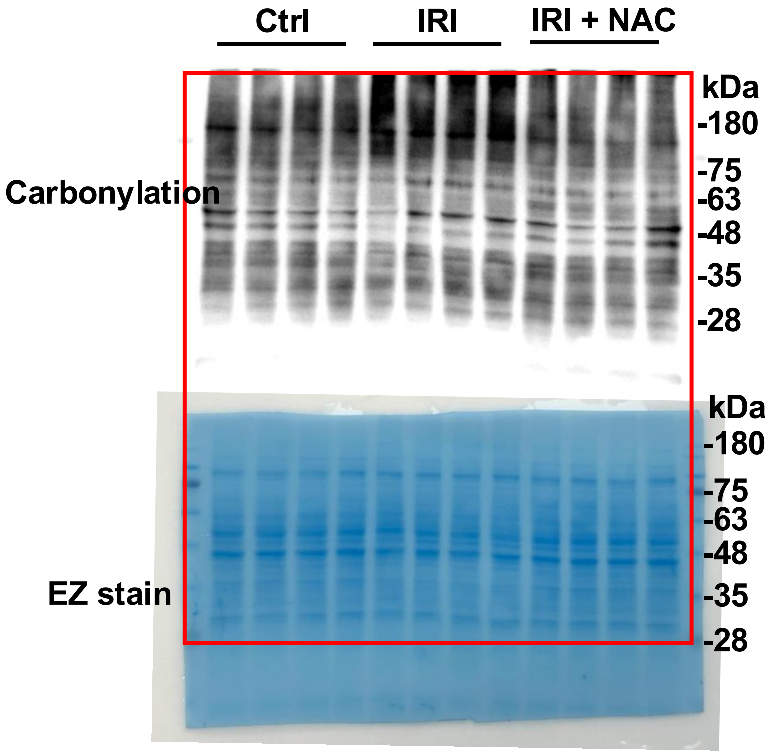

Fig 5C Kidney xCT & ACSL4

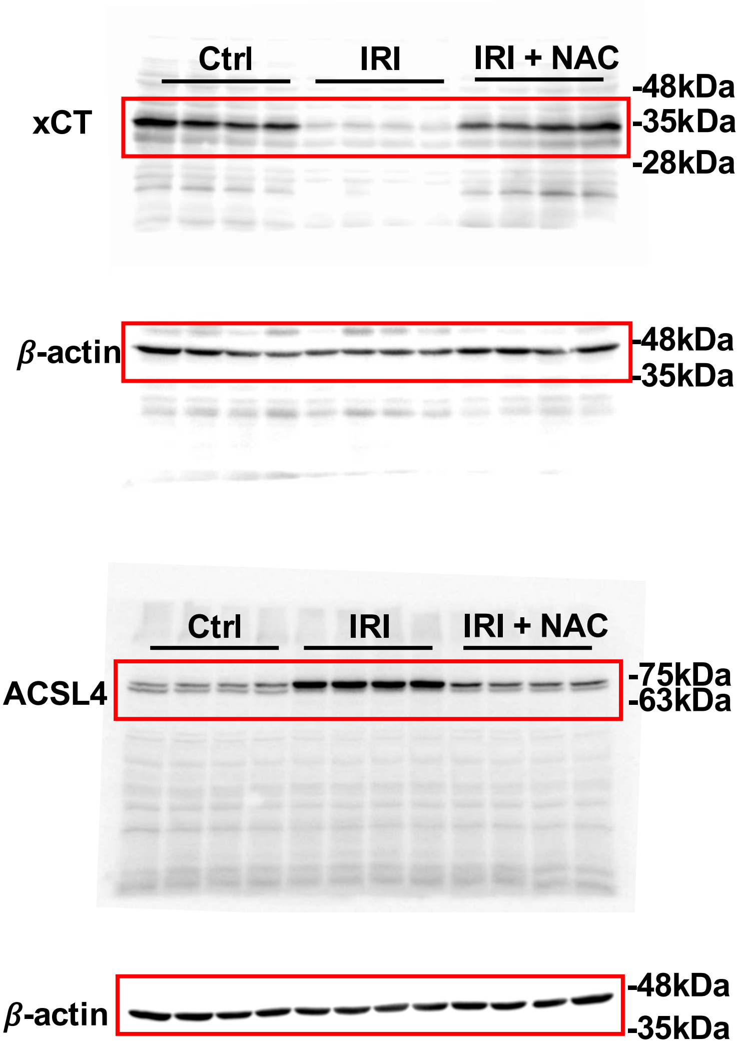

Fig 5F Colon -SH

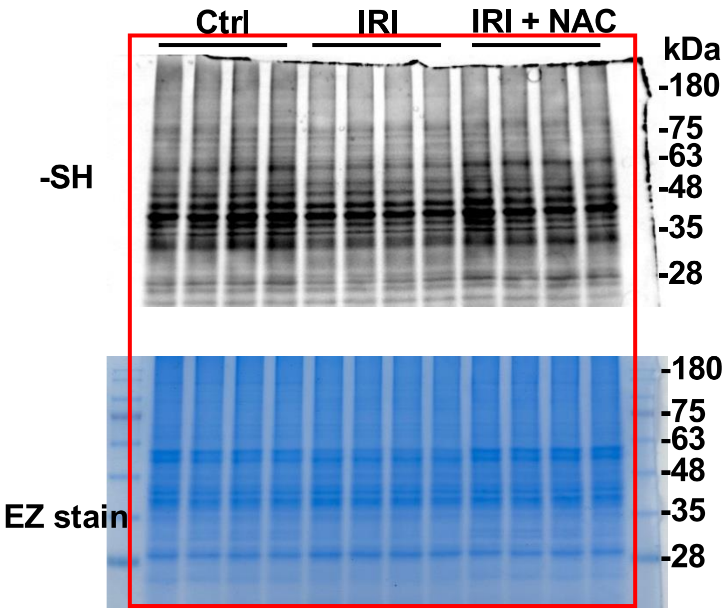

Fig 5G Colon -carbonylation

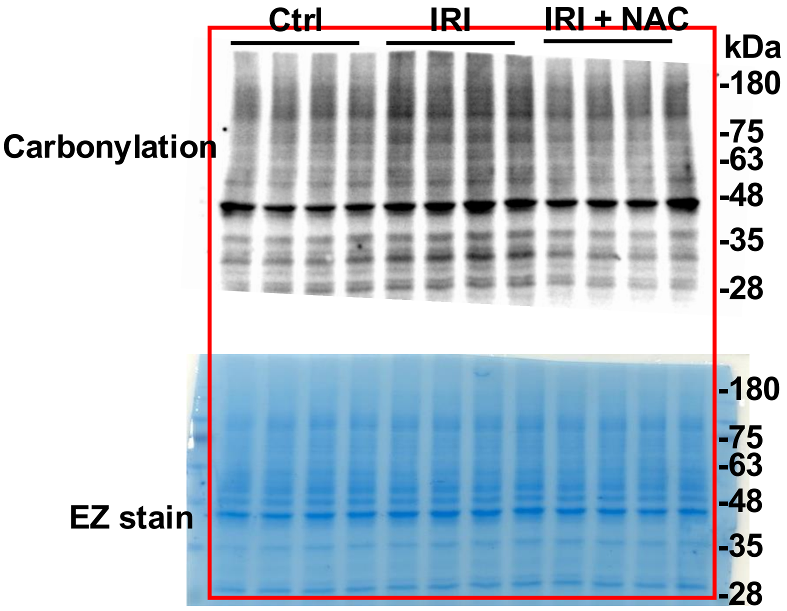

Fig 5H Serum -SH

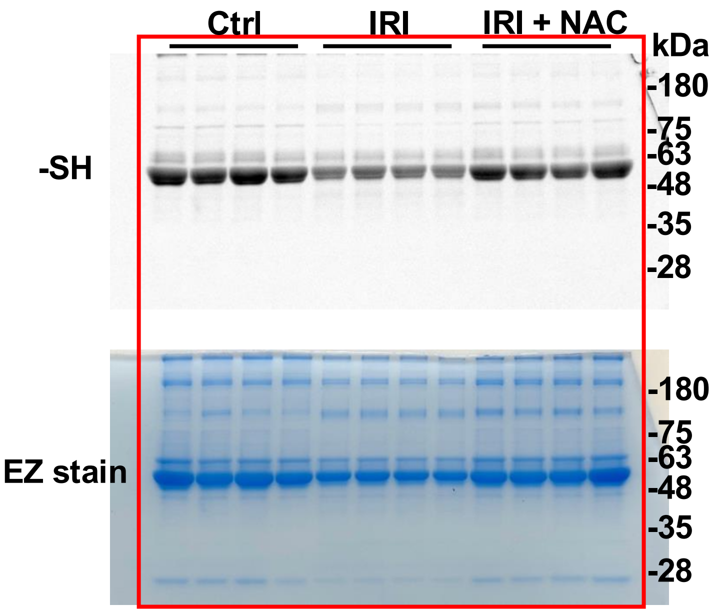

Fig 5I Serum -carbonylation

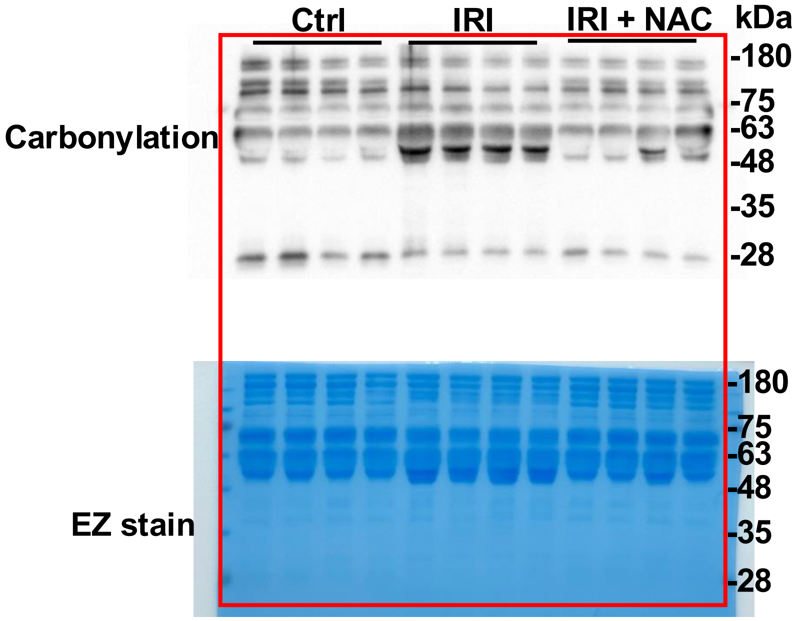

Fig 6A SH & carbonylation

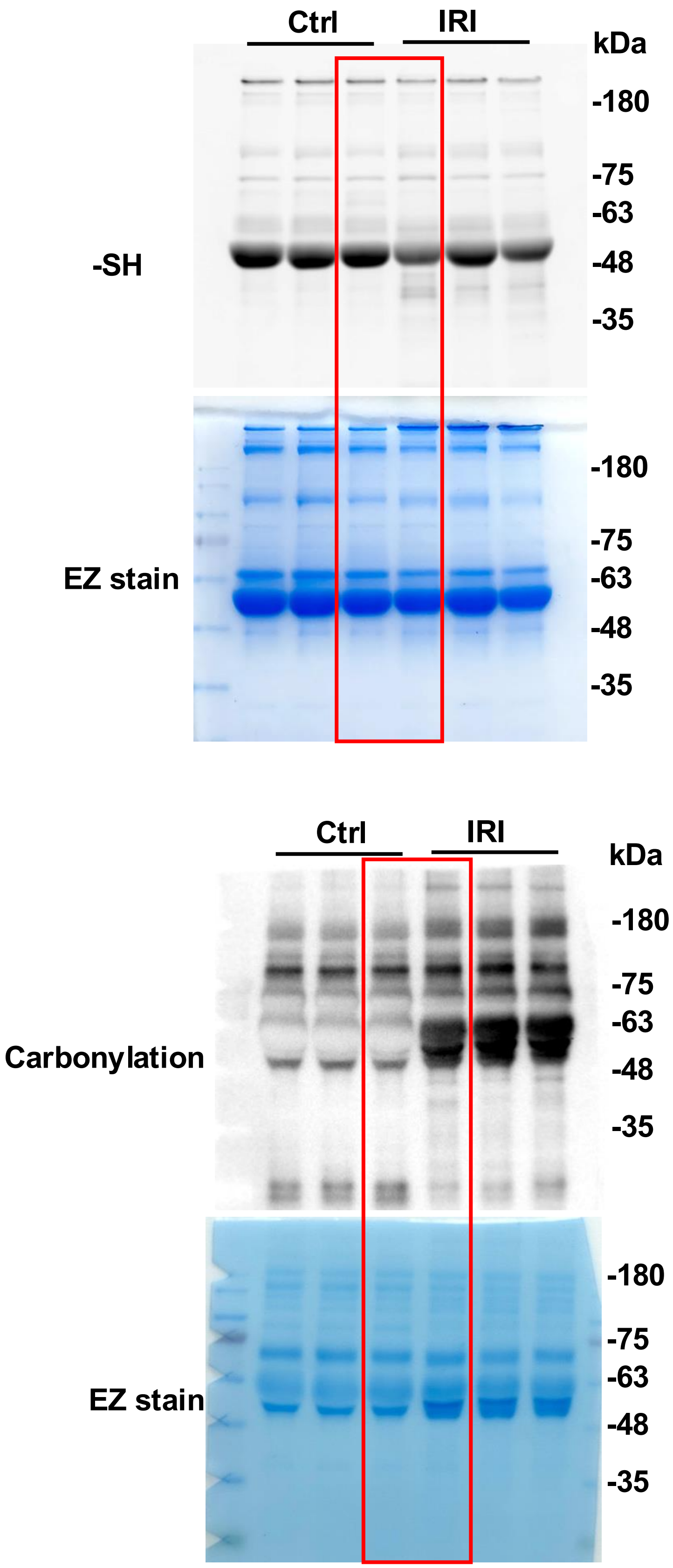

Fig 6D E-cadherin

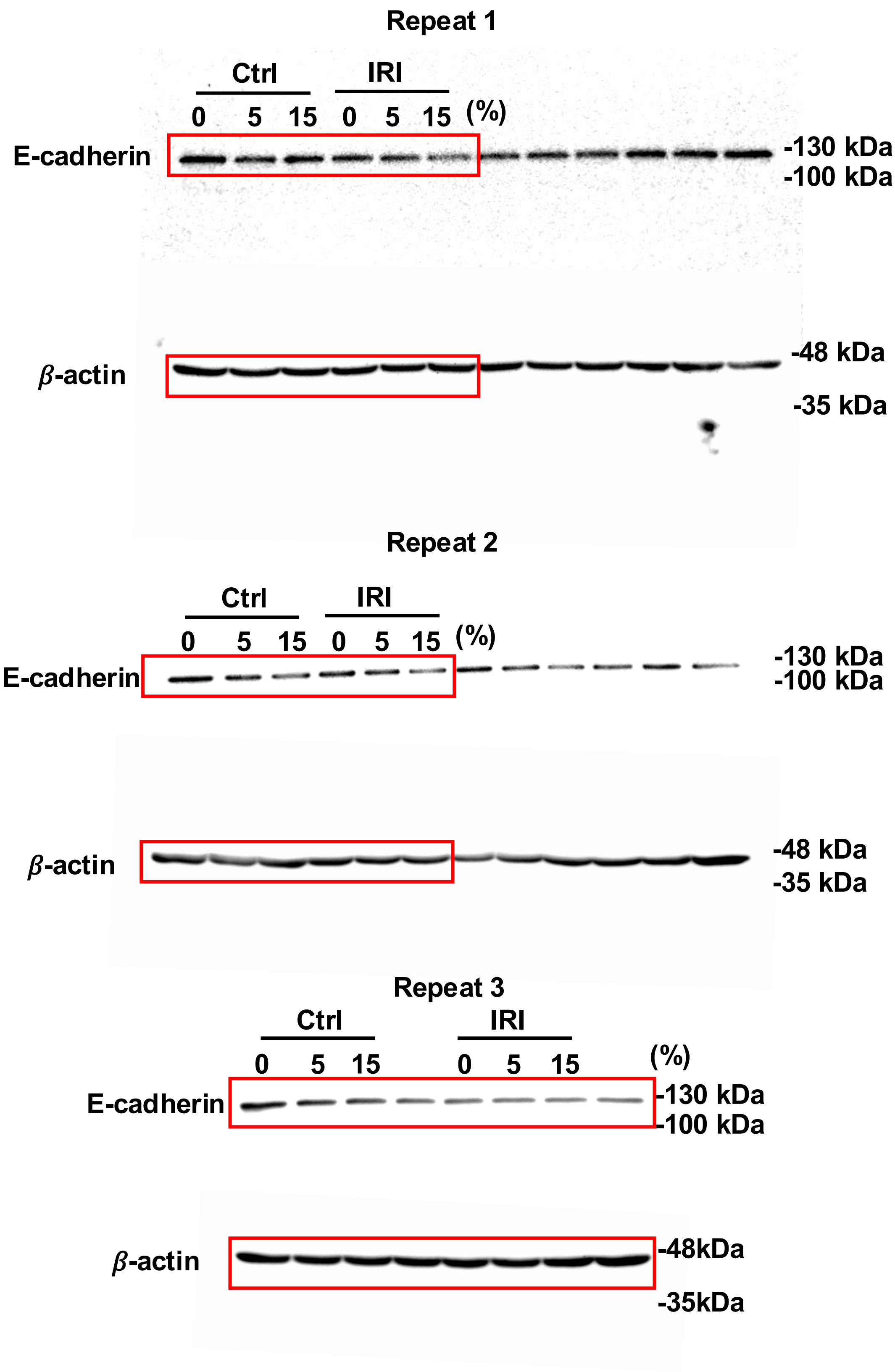

Fig 7A SH & carbonylation

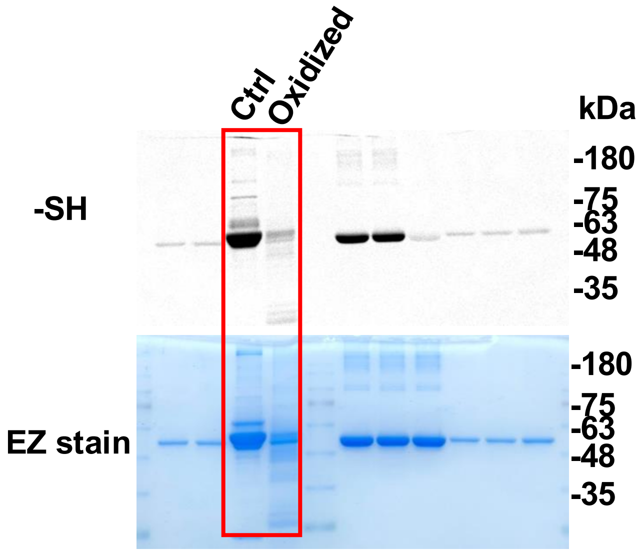

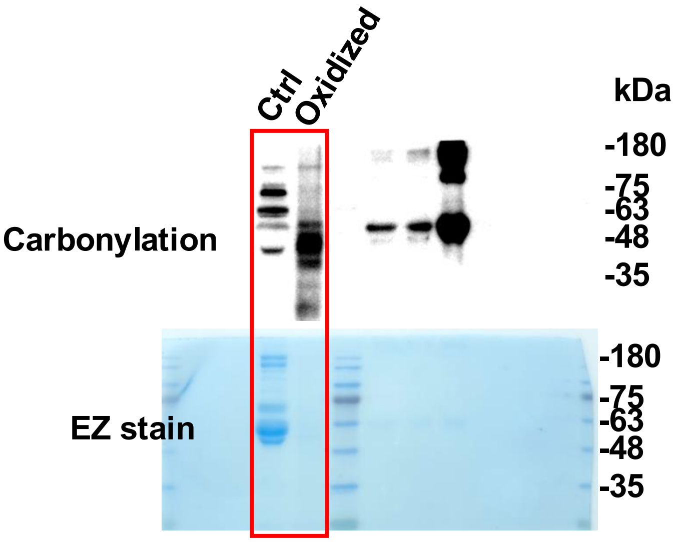

Fig 7D E-cadherin

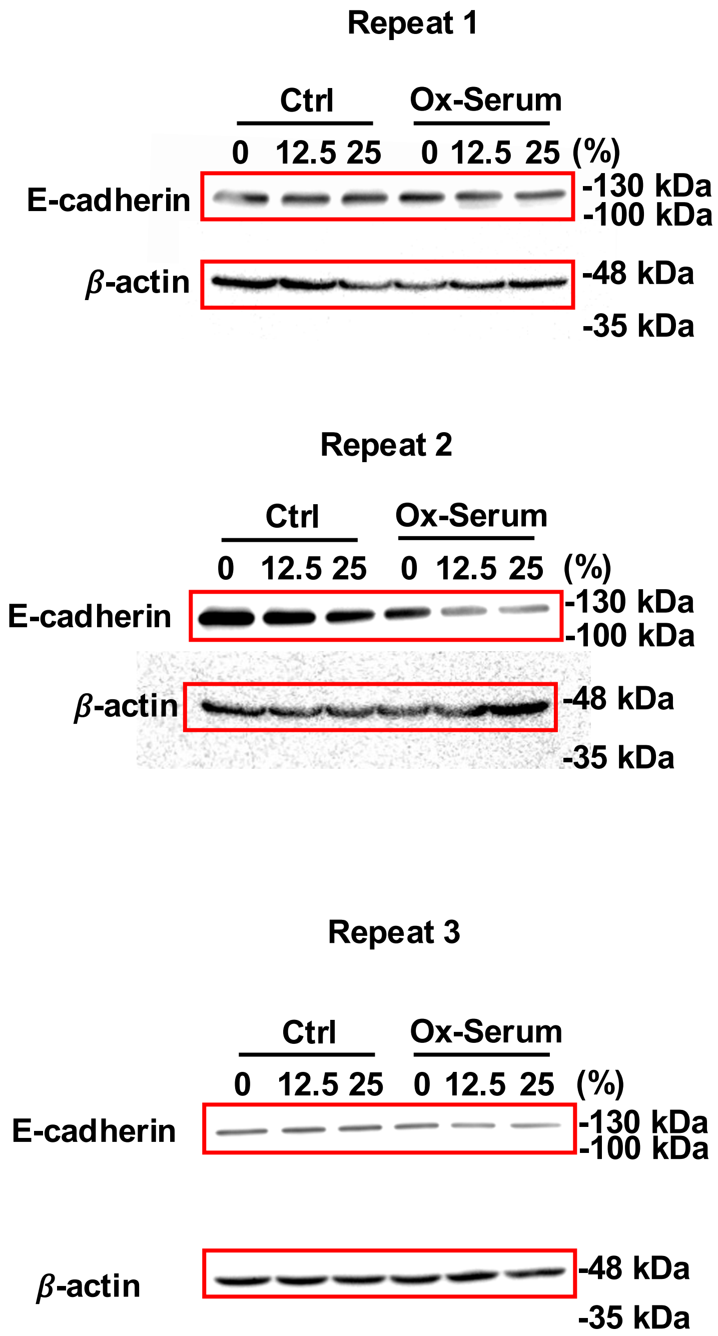

Fig 8A depletion albumin

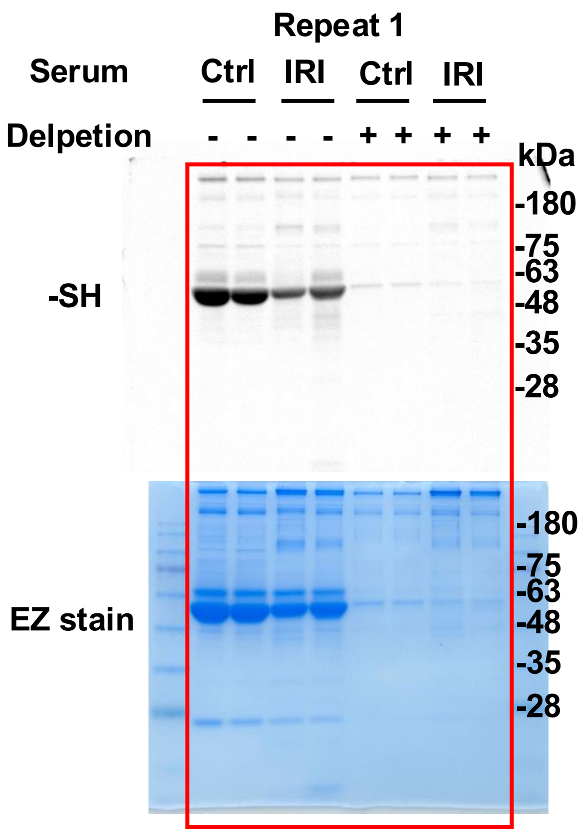

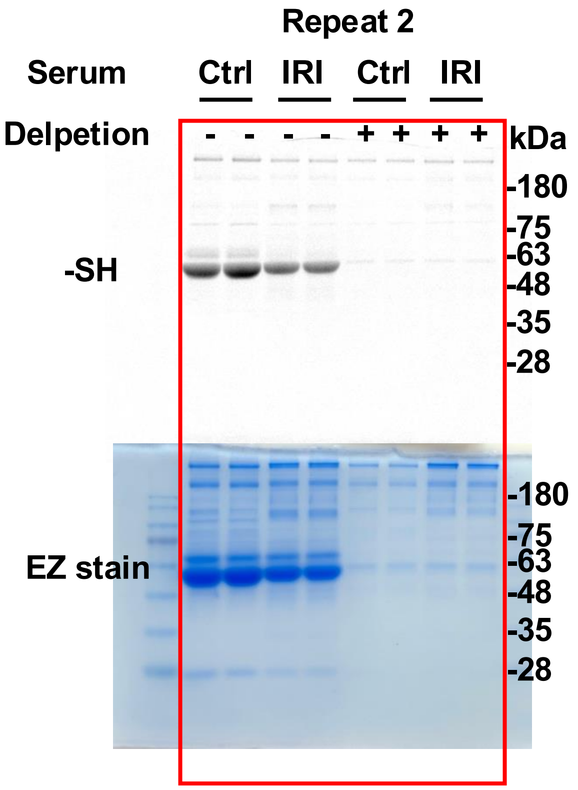

Fig 8D Albumin SH & carbonylation

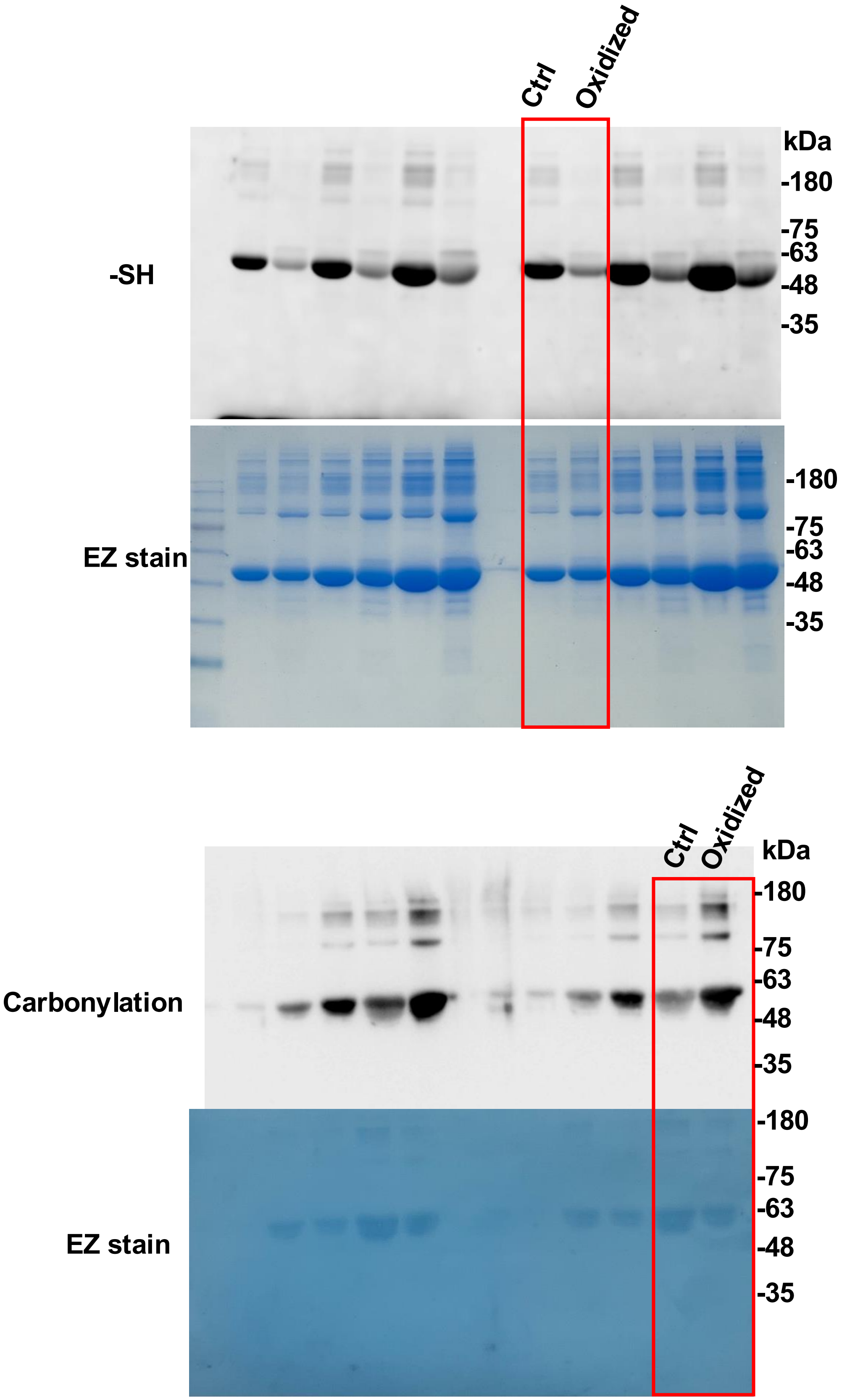

Supplement: Supplementary file 1 [file biomolecules-16-00462-s001.zip › biomolecules-4163782-supplementary.pdf]
